# Association of obesity under different metabolic status with adverse outcomes in patients with chronic myeloid leukemia: A retrospective cohort study

**DOI:** 10.1111/1753-0407.13383

**Published:** 2023-04-28

**Authors:** Yingchun Dong, Yiping Cheng, Xiaoshan Feng, Zinuo Yuan, Hang Dong, Yue Zhang, Junming Han, Yafei Wu, Zhixiang Wang, Xia Zhong, Xiude Fan, Jiajun Zhao

**Affiliations:** ^1^ Department of Endocrinology, Shandong Provincial Hospital Shandong University Jinan China; ^2^ Department of Endocrinology Shandong Provincial Hospital Affiliated to Shandong First Medical University Jinan China; ^3^ Shandong Clinical Research Center of Diabetes and Metabolic Diseases Jinan China; ^4^ Shandong Institute of Endocrine and Metabolic Diseases Jinan China; ^5^ Shandong Engineering Laboratory of Prevention and Control for Endocrine and Metabolic Diseases Jinan China; ^6^ Shandong Engineering Research Center of Stem Cell and Gene Therapy for Endocrine and Metabolic Diseases Jinan China; ^7^ Department of General Practice Shandong Provincial Hospital Affiliated to Shandong First Medical University Jinan China

**Keywords:** adverse outcomes, chronic myeloid leukemia, metabolic abnormalities, obesity, 不良结局, 慢性粒细胞白血病, 代谢异常, 肥胖

## Abstract

**Background:**

Little is known about the association between abnormal metabolic obesity states and the outcomes of chronic myeloid leukemia (CML), especially in patients with obesity with different metabolic status. Here, we used the Nationwide Readmissions Database to assess the effects of metabolically defined obesity on adverse outcomes of CML.

**Methods:**

Of the 35 460 557 (weighted) patients, we included 7931 adults with discharge diagnoses of CML from January 1, 2018 to June 30, 2018. The study population was observed until December 31, 2018 and divided into four groups based on body mass index and metabolic status. The primary outcome was the adverse outcomes of CML, including nonremission (NR)/relapse and severe mortality risk. Multivariate logistic regression analysis was performed to analyze data.

**Results:**

Metabolically unhealthy normal weight and metabolically unhealthy obesity were all risk factors for adverse outcomes of CML compared with metabolically healthy normal weight (all *p* < 0.01), and a significant difference was not found in the metabolically healthy obese. Female patients with metabolically unhealthy normal weight and metabolically unhealthy obesity had 1.23‐fold and 1.40‐fold increased NR/relapse risk, while male patients did not have this risk. Moreover, patients with a higher number of metabolic risk factors or with dyslipidemia were at higher risk of adverse outcomes, regardless of obesity status.

**Conclusions:**

Metabolic abnormalities were associated with adverse outcomes in patients with CML, irrespective of obesity status. Future treatment of patients with CML should consider the effects of obesity on their adverse outcomes under different metabolic status, especially in female patients.

## Graphical abstract

**Highlights**

- To our knowledge, our study is the first to date to investigate the association between metabolically defined obesity and adverse outcomes in patients with chronic myeloid leukemia (CML).
- Metabolic abnormalities were associated with adverse outcomes in patients with CML, irrespective of obesity status.
- There were sex modifications in metabolically defined obesity and the nonremission/relapse risk of CML.

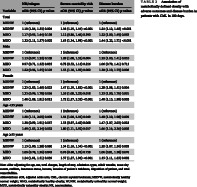

## 摘要

**目的:**目前对异常代谢性肥胖状态与慢性粒细胞白血病(CML)预后的关系知之甚少, 尤其是不同代谢状态的肥胖患者。在本研究中, 我们利用全国再入院数据库评估了代谢定义的肥胖对CML不良结局的影响。

**方法:**纳入2018年1月1日至2018年6月30日出院诊断为CML的成人患者7931例。研究人群观察至2018年12月31日, 根据体重指数和代谢状态分为4组。主要结局指标为CML的不良结局, 包括未缓解(NR)/复发和严重死亡风险。采用多因素logistic回归分析数据。

**结果:**与代谢健康的正常体重患者相比, 代谢不健康的正常体重和代谢不健康的肥胖均是CML不良结局的危险因素(均p < 0.01), 而代谢健康的肥胖与CML不良结局差异无统计学意义。代谢不健康的正常体重女性患者和代谢不健康的肥胖患者的NR/复发风险分别增加1.23倍和1.40倍, 而男性患者则无明显变化。此外, 无论肥胖状态如何, 合并代谢危险因素数量越多或血脂异常的患者发生不良结局的风险越高。

**结论:**无论肥胖是否处于健康还是不健康状态, 代谢异常与CML患者不良预后相关。未来CML患者的治疗应考虑不同代谢状态下肥胖对其不良结局的影响, 尤其是女性患者。

## 1 INTRODUCTION

Chronic myeloid leukemia (CML), a clonal hematopoietic stem cell malignancy, is characterized by unique translocation leading to the formation of the Philadelphia chromosome.^1^ Because of the specific genotype knowledge, tyrosine kinase inhibitors have been used in CML and have significantly improved the prognosis of patients with CML, especially in high‐income countries like the United States.^2^, ^3^ Notably, the 5‐year survival rates increased from 40% to 90%. So, CML is increasingly seen as a chronic instead of a potentially fatal disease.^4^ Previous studies showed that 30% of patients with CML were obese and had hypertension, 11% had diabetes, and 18% had dyslipidemias,^5^ all of which might lead to relapse of CML and death and create a huge burden on individuals, families, and countries. However, the influence of obesity and metabolic abnormalities as modifiable factors on CML is not clear yet.

Obesity is increasingly prevalent worldwide and is considered to be an important risk factor for adverse outcomes in many cancers.^6^ A prospective study of more than 900 000 US adults found that overweight and obesity account for 14% of all cancer deaths in men and 20% of those in women.^7^ Obesity is well‐known for being associated with metabolic abnormalities like hyperglycemia, hypertension, or dyslipidemia.^8^ The metabolic abnormalities and obesity‐induced systemic chronic inflammation contribute to tumorigenesis and progression.^9^ However, patients with obesity with CML had a better outcome than patients with normal body weight,^10^, ^11^ which might be an “obesity paradox.”^12^ Interestingly, some reports have also illustrated that a minority of individuals with obesity do not have metabolic abnormalities.^13^, ^14^ Obesity alone may not reflect the actual metabolic health state, and it appears to be more accurate to reclassify patients based on the combination of body mass index (BMI) and metabolic status to further determine the relationship between obesity itself or the combination of metabolic status and prognosis of patients with CML.

To the best of our knowledge, it is uncertain whether metabolic status plays a role in the prognosis of patients with CML. In addition, no previous studies have investigated the association between metabolically defined obesity and outcomes in patients with CML. Therefore, we conducted a retrospective cohort study using the Nationwide Readmissions Database (NRD) to explore the impact of metabolically defined obesity on the adverse outcomes in patients with CML, which benefits more reliable identification of high‐risk populations and effective prevention and treatment strategies.

## 2 METHODS

### 2.1 Data sources and study population

Of the 35 460 557 (weighted) patients, we identified 23 008 patients diagnosed with CML in the 2018 NRD database using the *International Classification of Diseases, Tenth Revision, Clinical Modification* (ICD‐10‐CM) diagnostic (Table S1 in Data S1). The 2018 NRD database contains information from 28 states that represent 60% of the US population.^15^ The characteristics of the national readmission for patients can be studied based on a verified unique linkage number that enables tracking of patients across different hospitals within a state but does not across years.^15^ Meantime, it was publicly available and one of the largest inpatient national sample databases. The study was based on STROBE reporting guidelines and abided by the United States Agency for Healthcare Research and Quality's Healthcare Cost and Utilization Project Data Use Agreement and was exempt from research ethics board review. Consent to participate was not applicable because the data were extracted from the 2018 NRD database. The data were available from the Healthcare Cost and Utilization Project site, subject to a registration and application process.

We excluded patients who had missing baseline characteristic data (*n* = 245), who were aged <18 years (*n* = 103), who were pregnant (*n* = 71), who died during the initial hospitalization (*n* = 638), who had low body weight (BMI ≤ 19.9 kg/m^2^, *n* = 371), and who were initially hospitalized between July 1, 2018 and December 31, 2018 for 180 days (*n* = 6043, Figure S1). Finally, we included 7931 adults with discharge diagnoses of CML from January 1, 2018 to June 30, 2018. The ICD‐10‐CM codes used are shown in Table S1 in Data S1.

### 2.2 Exposure measures

Patients were classified into two BMI categories: normal weight (BMI < 25 kg/m^2^) and obesity (BMI ≥ 25 kg/m^2^). Metabolic abnormalities were defined as having any two of the following according to the harmonized International Diabetes Federation criteria^16^: (1) hyperglycemia (including prediabetes, diabetes, and others), (2) dyslipidemia (including hypertriglyceridemia, hypercholesterolemia, and others), and (3) hypertension. The definition of metabolic abnormalities excluded waist circumference because of its collinearity with BMI. Patients were classified into four groups according to BMI categories and metabolic status: (1) metabolically healthy normal weight (MHNW); (2) metabolically unhealthy normal weight (MUNW); (3) metabolically healthy obesity (MHO); and (4) metabolically unhealthy obesity (MUO). The above diagnoses were determined by ICD‐10‐CM codes (Table S2 in Data S1).

To explore the impact of the number of metabolic risk factors and specific metabolic risk factors on adverse outcomes of CML, patients were classified into eight groups based on BMI and the number of metabolic risk factors: (1) normal weight with no metabolic risk factors; (2) normal weight with one metabolic risk factor; (3) normal weight with two metabolic risk factors; (4) normal weight with three metabolic risk factors; (5) obesity with no metabolic risk factors; (6) obesity with one metabolic risk factor; (7) obesity with two metabolic risk factors; and (8) obesity with three metabolic risk factors. In addition, patients were classified into eight groups based on BMI and specific metabolic risk factors: (1) normal weight with no metabolic risk factors; (2) normal weight only with hyperglycemia; (3) normal weight only with hypertension; (4) normal weight only with hyperlipidemia; (5) obesity with no metabolic risk factors; (6) obesity only with hyperglycemia; (7) obesity only with hypertension; and (8) obesity only with hyperlipidemia.

### 2.3 Outcome measures

The primary outcome was the adverse outcomes (nonremission [NR]/relapse and severe mortality risk) in patients with CML on different readmission days. Readmission was defined as diagnoses with CML and unplanned hospital readmissions from the discharge, and we only recorded the first readmission for patients with multiple readmissions.^17^ The NR/relapse in patients with CML was diagnosed by using ICD‐10‐CM. Severe mortality risk was defined as the major likelihood of dying based on the risk‐of‐mortality subclass in the 2018 NRD database.^15^ Disease burden was defined as hospital readmission costs due to illness. Severity of illness was defined as major or extreme loss of function based on the severity‐of‐illness subclass in the 2018 NRD database.^15^ Secondary outcome included disease burden, severity of illness, further analysis of the impact of the number of metabolic risk factors, and specific metabolic risk factors on the adverse outcomes in patients with CML.

### 2.4 Variables

We identified baseline characteristics of study participants using NRD variables such as age, sex, total charges, length of stay, admission types (whether nonelective), rehab transfer, same‐day events (transfer flag indicating a combination of discharges involving same‐day events), resident, insurance status (Medicare, Medicaid, private insurance, self‐pay, no charge, or other), median household income by ZIP code, location of patient's residence, disposition of patient, hyperglycemia, hypertension, dyslipidemia, severity of illness, severe mortality risk, and comorbidities (e.g., ischemic heart disease, chronic obstructive pulmonary disease, neurologic condition, chronic kidney disease, and other oncologic diseases).^18^ The required ICD‐10‐CM codes are given in Table S2 in Data S1.

### 2.5 Statistical analysis

We summarized the baseline characteristics in patients with metabolically defined obesity. Frequency counts and percentages were appropriate for categorical variables and were compared using the chi‐square test, whereas medians (interquartile range [IQR]) were used for continuous variables with non‐normal distribution and uneven variances and were compared using the Kruskal–Wallis *H* test. Multivariable logistic regression was performed to analyze data. Results were denoted as odds ratios (ORs) with 95% CI. Discharge weights were used to generate national estimates. All hypothesis tests were two‐sided, and a *p* < 0.05 was deemed statistically significant. All statistical analyses were performed using the SPSS software 26.0.

### 2.6 Sensitivity analysis

To evaluate the robustness of findings, the following sensitivity analyses were performed. We did the same analysis at 90 and 30 days (Figure S1).

## 3 RESULTS

### 3.1 Baseline characteristics of patients with CML with metabolically defined obesity

Based on our inclusion and exclusion criteria, we included 7931 (56.8%) hospital discharges for adult patients for the 180‐day readmission analysis (MHNW [3585, 45.2%], MUNW [3060, 38.6%], MHO [525, 6.6%], MUO [761, 9.6%]), 10 980 (78.6%) discharges for the 90‐day analysis (MHNW [4946, 45.0%], MUNW [4218, 38.4%], MHO [748, 6.8%], MUO [1068, 9.7%]), and 12 953 (92.7%) discharges for the 30‐day analysis (MHNW [5801, 44.8%], MUNW [5040, 38.9%], MHO [878, 6.8%], MUO [1234, 9.5%]) (Figure S1). Baseline characteristics of study participants are summarized in Table 1. The average age was 68 years and males accounted for 56.7%. The patients with MUNW or MUO were older and had higher mortality risk compared to the patients with MHNW or MHO (all *p* < 0.01), meaning that patients with metabolic abnormalities were older and seemed to be at a higher mortality level. Moreover, patients with MHO or MUO, representing patients with obesity, had more hospitalization costs and longer lengths of stay (all *p* < 0.01). Compared with MHNW, the patients with MUNW, MHO, and MUO had higher severity of illness (all *p* < 0.01). Baseline characteristics of study participants were generally similar between the different readmission days (Tables S3 and S4 in Data S1).

**TABLE 1:** Baseline characteristics of patients with CML with metabolically defined obesity in 180 days.

Variable	Total (*n* = 7931)	MHNW (*n* = 3585)	MUNW (*n* = 3060)	MHO (*n* = 525)	MUO (*n* = 761)	*p* value
Age (years) median (IQR)	68 (57, 78)	64 (51, 77)^a^	74 (65, 81)^b^	58 (46, 67)^c^	66 (57, 74)^a^	<0.001
Age ≥55 years, *n* (%)	6104 (77)	2422 (67.6)^a^	2783 (90.9)^b^	302 (57.4)^c^	597 (78.4)^d^	<0.001
Male, *n* (%)	4496 (56.7)	1998 (55.7)^a^	1808 (59.1)^b^	266 (50.6)^a^	424 (55.6)^a,b^	0.001
Total charges ($) median (IQR)	38792.5 (21324.0, 75235.55)	36582.9 (20808.0, 74833.0)^a^	39310.0 (21128.0, 72801.82)^a^	45156.34 (25487.0, 78057.03)^b^	40864.95 (23136.67, 84133.78)^b^	<0.001
Length of stay (days) median (95% CI)	4.00 (6.19, 6.60)	4.00 (6.39, 7.05)^a^	4.00 (5.44, 5.89)^a^	5.00 (6.92, 8.35)^b^	4.00 (5.87, 8.02)^c^	<0.001
Nonelective, *n* (%)	6885 (86.8)	3118 (87.0)^a^	2682 (87.6)^a^	441 (83.8)^a^	644 (846.)^a^	0.027
Rehab transfer, *n* (%)	119 (1.5)	61 (1.7)^a^	46 (1.5)^a^	2 (0.4)^a^	10 (1.3)^a^	0.132
Same‐day events, *n* (%)	375 (4.7)	175 (4.9)^a^	133 (4.3)^a^	30 (5.7)^a^	37 (4.9)^a^	0.509
Resident, *n* (%)	7393 (93.2)	3332 (93.0)^a,b^	2861 (93.5)^a,b^	477 (90.9)^b^	723 (94.9)^a^	0.034
Insurance status, *n* (%)						<0.001
Medicare	5223 (65.9)	2012 (56.1)^a^	2401(78.5)^b^	264 (50.2)^a^	546 (71.8)^c^	
Medicaid	741 (9.3)	483 (13.5)^a^	132 (4.3)^b^	72 (13.7)^a^	54 (7.1)^c^	
Private insurance	1679 (21.2)	918 (25.6)^a^	448 (14.6)^b^	165 (31.4)^c^	148 (19.5) ^d^	
Self‐pay	117 (1.5)	88 (2.5)^a^	16 (0.5)^b^	9 (1.7)^a,c^	4 (0.5)^b,c^	
No charge	17 (0.2)	9 (0.3)^a^	3 (0.1)^a^	2 (0.4)^a^	3 (0.4)^a^	
Other	154 (1.9)	75 (2.1)^a^	60 (2.0)^a,b^	14 (2.7)^a^	5 (0.7)^b^	
Median household income by ZIP code, *n* (%)						0.001
$1–$45 999	2027 (25.6)	923 (25.8)^a^	779 (25.5)^a^	125 (23.8)^a^	200 (26.2)^a^	
$46 000–$58 999	2453 (30.9)	1106 (30.9)^a^	886 (29.0)^a^	179 (34.1)^a,b^	282 (37.0)^b^	
$59 000–$78 999	1869 (23.6)	843 (23.5)^a,b^	748 (24.4)^b^	130 (24.8)^a,b^	148 (19.4)^a^	
> $79 000	1582 (19.9)	712 (19.9)^a^	647 (21.1)^a^	91 (17.3)^a^	132 (17.3)^a^	
Location of patient's residence, *n* (%)						<0.001
Large central counties	1849 (23.3)	906 (25.3)^a^	682 (22.3)^b^	85 (16.2)^c^	176 (23.1)^a,b^	
Large fringe counties	2163 (27.3)	947 (26.4)^a,b^	882 (28.8)^b^	159 (30.3)^b^	175 (23.0)^a^	
Medium metro counties	1739 (21.9)	736 (20.5)^a^	673 (22.0)^a,b^	138 (26.3)^b^	192 (25.2)^b^	
Small metro counties	808 (10.2)	376 (10.5)^a^	291 (9.5)^a^	49 (9.4)^a^	92 (12.1)^a^	
Micropolitan counties	829 (10.5)	395 (11.0)^a^	308 (10.1)^a^	46 (8.8)^a^	80 (10.5)^a^	
Not metro/micropolitan counties	541 (6.8)	224 (6.3)^a^	223 (7.3)^a^	47 (9.0)^a^	47 (6.2)^a^	
Disposition of patient, *n* (%)						<0.001
Routine	4982 (62.8)	2384 (66.5)^a^	1787 (58.4)^b^	366 (69.7)^a^	445 (58.4)^b^	
Transfer to short‐term hospital	93 (1.2)	39 (1.1)^a^	30 (1.0)^a^	13 (2.5)^b^	11 (1.4)^a,b^	
Transfer other	1222 (15.4)	464 (12.9)^a^	570 (18.6)^b^	52 (9.9)^a^	136 (17.8)^b^	
Home health care	1553 (19.6)	641 (17.9)^a^	658 (21.5)^b^	89 (17.0)^a,b^	165 (21.7)^a,b^	
Against medical advice	79 (1.0)	54 (1.5)^a^	15 (0.5)^b^	5 (1.0)^a,b^	5 (0.7)^a,b^	
Discharge alive, destination unknown	2 (0.0)	2 (0.1)^a^	0 (0.0)^a^	0 (0.0)^a^	0 (0.0)^a^	
Hyperglycemia, *n* (%)	2602 (32.8)	213 (5.9)^a^	1779 (58.1)^b^	67 (12.8)^c^	543 (71.3) ^d^	<0.001
Hypertension, *n* (%)	5440 (68.6)	1496 (41.7)^a^	2961 (96.8)^b^	252 (47.9)^c^	731 (95.9)^b^	<0.001
Dyslipidemia, *n* (%)	3273 (41.3)	292 (8.1)^a^	2393 (78.2)^b^	32 (6.1)^a^	556 (73.1)^c^	<0.001
Major or extreme loss of function, *n* (%)	4687 (59.1)	1884 (52.6)^a^	1927 (63.0)^b^	325 (61.9)^b^	551 (72.3)^c^	<0.001
Risk of severe mortality, *n* (%)	3456 (43.6)	1292 (36.0)^a^	1571(51.3)^b^	202 (38.5)^a^	391 (51.3)^b^	<0.001
Comorbidities						
Ischemic heart disease, *n* (%)	136 (1.7)	31 (0.9)^a^	74 (2.4)^b^	9 (1.7)^a,b^	22 (2.9)^b^	<0.001
Chronic obstructive pulmonary disease, *n* (%)	129 (1.6)	55 (1.5)^a,b^	52 (1.7)^a,b^	2 (0.4)^b^	20 (2.6)^a^	0.017
Neurologic condition, *n* (%)	347 (4.4)	163 (4.5)^a^	132 (4.3)^a^	17 (3.2)^a^	35 (4.6)^a^	0.569
Chronic kidney disease, *n* (%)	352 (4.4)	82 (2.3)^a^	201 (6.6)^b^	10 (1.9)^a^	59 (7.7)^b^	<0.001
Female reproductive malignancy, *n* (%)	39 (0.5)	15 (0.4)^a^	19 (0.6)^a^	1 (0.2)^a^	4 (0.5)^a^	0.488
Digestive system malignancy, *n* (%)	74 (0.9)	36 (1.0)^a^	29 (0.9)^a^	0 (0.0)^a^	9 (1.2)^a^	0.130
Primary pulmonary carcinoma, *n* (%)	65 (0.8)	28 (0.8)^a^	22 (0.7)^a^	9 (1.7)^a^	6 (0.8)^a^	0.131
Malignant tumor of urinary system, *n* (%)	113 (1.4)	48 (1.3)^a^	43 (1.4)^a^	13 (2.5)^a^	9 (1.2)^a^	0.202
Total comorbidities, *n* (%)						<0.001
<4	6986 (88.1)	3228 (90.1)^a^	2630 (85.9)^b^	482 (91.6)^a^	646 (84.8)^b^	

*Note*: Lowercase letters (e.g., a, b, c, d, etc.) refer to comparisons between groups. There is no statistical difference between groups with the same lowercase letters.

Abbreviations: CML, chronic myeloid leukemia; IQR, interquartile range; MHNW, metabolically healthy normal weight; MHO, metabolically healthy obesity; MUNW, metabolically unhealthy normal weight; MUO, metabolically unhealthy obesity.

### 3.2 Adverse outcomes and disease burden in patients with CML with metabolically defined obesity

**FIGURE 1:**
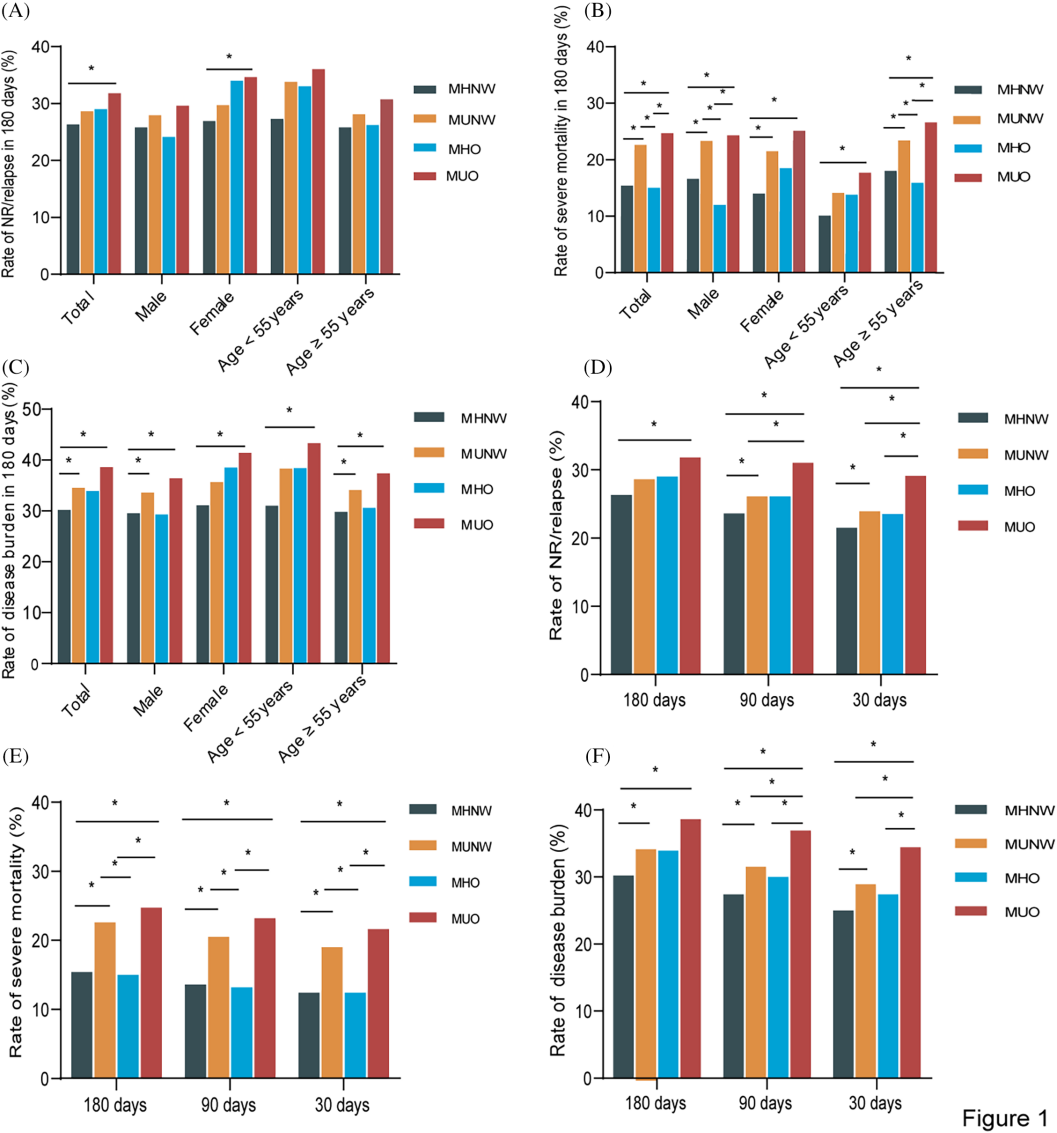
Rate of adverse outcomes and disease burden in patients with CML in metabolically defined obesity. (A) The rate of NR/relapse in 180 days. (B) The rate of severe mortality in 180 days. (C) The rate of disease burden in 180 days. (D) The rate of NR/relapse on different readmission days. (E) The rate of severe mortality on different readmission days. (F) The rate of disease burden on different readmission days. NR, non‐remission; MHNW, metabolically healthy normal weight; MUNW, metabolically unhealthy normal weight; MHO, metabolically healthy obesity; MUO, metabolically unhealthy obesity.

Patients with MUNW or MUO had a significantly higher rate of adverse outcomes and disease burden than those with MHNW (all *p* < 0.01), suggesting that patients with metabolic abnormalities were more likely to experience disease progression and death (Figure 1D,E,F). Interestingly, even though the rate of NR/relapse and rate of disease burden did not reach statistical significance in the MHO group, it seemed to be higher in the total, in females, and in patients aged <55 years than the MHNW, and similar to MUNW. Hence, obesity appeared to affect NR/relapse and disease burden at least in some groups (Figure 1A,C).

**FIGURE 2:**
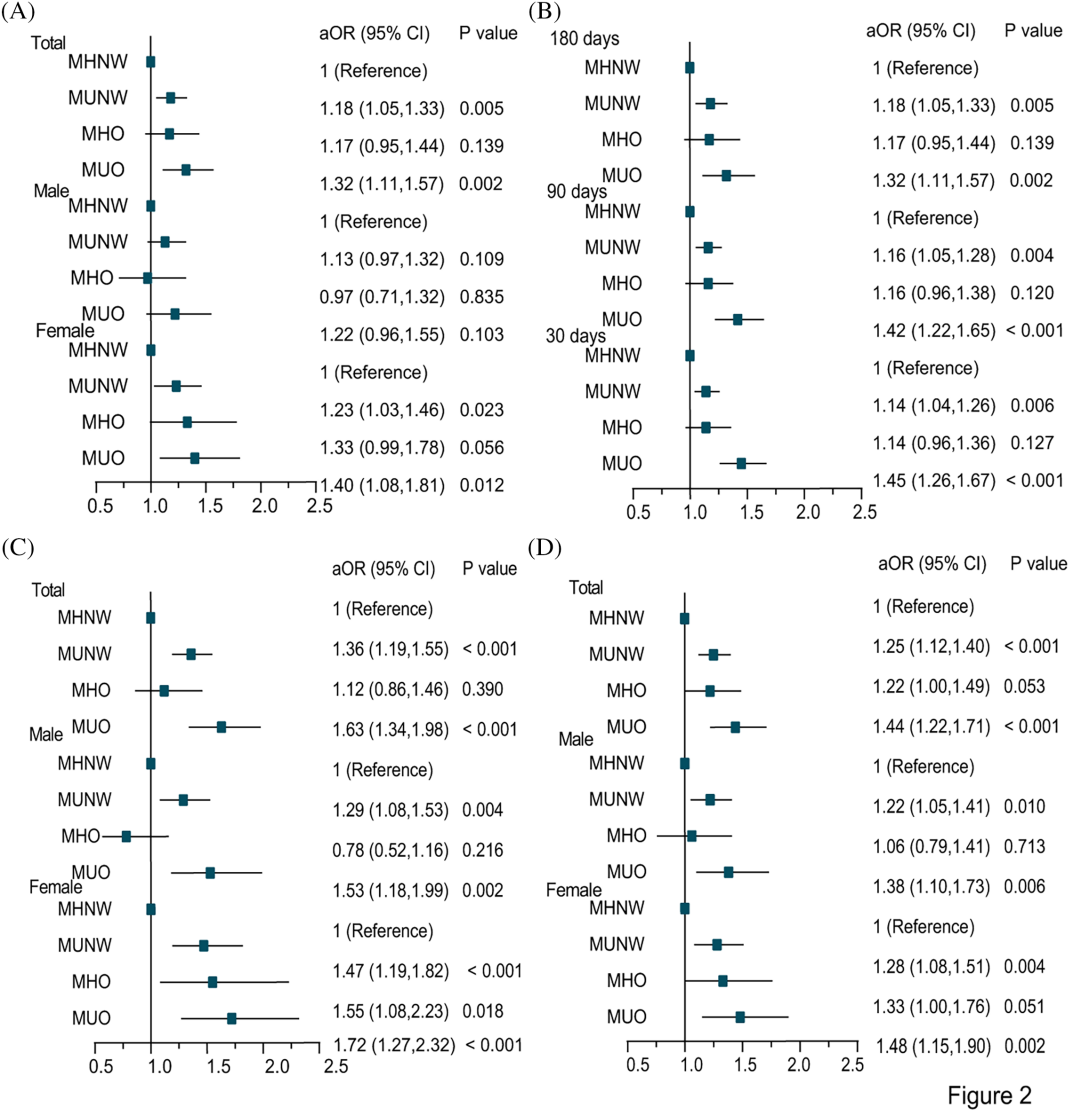
Forest plot of adverse outcomes and disease burden in patients with CML with metabolically defined obesity. (A) The NR/relapse in 180 days. (B) The NR/relapse on different readmission days. (C) The severe mortality risk in 180 days. (D) The disease burden in 180 days. NR, non‐remission; aOR, adjusted odds ratio; MHNW, metabolically healthy normal weight; MUNW, metabolically unhealthy normal weight; MHO, metabolically healthy obesity; MUO, metabolically unhealthy obesity.

After adjusting for age, sex, total charges, length of stay, admission types, rehab transfer, same‐day events, resident, insurance status, income, location of patient's residence, disposition of patient, and total comorbidities, patients with MUNW and MUO were associated with higher risk of NR/relapse (adjusted odds ratio [aOR] = 1.18, 95% CI, 1.05–1.33; aOR = 1.32, 95% CI, 1.11–1.57), severe mortality (aOR = 1.36, 95% CI, 1.19–1.56; aOR = 1.63, 95% CI, 1.34–1.98) and disease burden (aOR = 1.25, 95% CI, 1.12–1.40; aOR = 1.44, 95% CI, 1.22–1.71), but patients with MHO showed no significant differences in the risk of adverse outcomes and disease burden (Table 2). Sensitivity analyses with different readmission days had similar results (Figure 2B, Table S5 and S6 in Data S1). Interestingly, we found that patients with MHO were related to a higher risk of NR/relapse (aOR = 1.33, 95% CI, 0.99–1.78; aOR = 1.38, 95% CI, 0.99–1.91), severe mortality (aOR = 1.55, 95% CI, 1.08–2.23; aOR = 1.53, 95% CI, 0.97–2.42) and disease burden (aOR = 1.33, 95% CI, 1.00–1.76; aOR = 1.47, 95% CI, 1.07–2.03) in females and in patients aged <55 years compared with MHNW, even if sometimes there was no statistically significant difference in the multivariable logistic regression at 180 days, meaning that obesity seemed to affect adverse outcomes and disease burden in some subgroups (Table 2).

**TABLE 2:** Association of metabolically defined obesity with adverse outcomes and disease burden in patients with CML in 180 days.

Variable	NR/relapse	Severe mortality risk	Disease burden
	aOR (95% CI) *p* value	aOR (95% CI) *p* value	aOR (95% CI) *p* value
Total			
MHNW	1 (reference)	1 (reference)	1 (reference)
MUNW	1.18 (1.05, 1.33) 0.005	1.36 (1.19, 1.55) <0.001	1.25 (1.12, 1.40) <0.001
MHO	1.17 (0.95, 1.44) 0.139	1.12 (0.86, 1.46) 0.390	1.22 (1.00, 1.49) 0.053
MUO	1.32 (1.11, 1.57) 0.002	1.63 (1.34, 1.98) <0.001	1.44 (1.22, 1.71) <0.001
Male			
MHNW	1 (reference)	1 (reference)	1 (reference)
MUNW	1.13 (0.97, 1.32) 0.109	1.29 (1.08, 1.53) 0.004	1.22 (1.05, 1.41) 0.010
MHO	0.97 (0.71, 1.32) 0.835	0.78 (0.52, 1.16) 0.216	1.06 (0.79, 1.41) 0.713
MUO	1.22 (0.96, 1.55) 0.103	1.53 (1.18, 1.99) 0.002	1.38 (1.10, 1.73) 0.006
Female			
MHNW	1 (reference)	1 (reference)	1 (reference)
MUNW	1.23 (1.03, 1.46) 0.023	1.47 (1.19, 1.82) <0.001	1.28 (1.08, 1.51) 0.004
MHO	1.33 (0.99, 1.78) 0.056	1.55 (1.08, 2.23) 0.018	1.33 (1.00, 1.76) 0.051
MUO	1.40 (1.08, 1.81) 0.012	1.72 (1.27, 2.32) <0.001	1.48 (1.15, 1.90) 0.002
Age <55 years			
MHNW	1 (reference)	1 (reference)	1 (reference)
MUNW	1.50 (1.11, 2.03) 0.009	1.54 (1.00, 2.36) 0.049	1.48 (1.10, 1.98) 0.009
MHO	1.38 (0.99, 1.91) 0.057	1.53 (0.97, 2.42) 0.068	1.47 (1.07, 2.03) 0.018
MUO	1.49 (1.03, 2.14) 0.032	1.80 (1.11, 2.92) 0.017	1.66 (1.16, 2.36) 0.005
Age ≥55 years			
MHNW	1 (reference)	1 (reference)	1 (reference)
MUNW	1.13 (1.00, 1.28) 0.060	1.34 (1.16, 1.54) <0.001	1.20 (1.07, 1.36) 0.003
MHO	1.03 (0.78, 1.36) 0.845	0.95 (0.68, 1.33) 0.758	1.06 (0.81, 1.38) 0.676
MUO	1.24 (1.01, 1.51) 0.039	1.57 (1.27, 1.96) <0.001	1.35 (1.11, 1.63) 0.002

*Note*: After adjusting for age, sex, total charges, length of stay, admission types, rehab transfer, same‐day events, resident, insurance status, income, location of patient's residence, disposition of patient, and total comorbidities.

Abbreviations: aOR, adjusted odds ratio; CML, chronic myeloid leukemia; MHNW, metabolically healthy normal weight; MHO, metabolically healthy obesity; MUNW, metabolically unhealthy normal weight; MUO, metabolically unhealthy obesity; NR, nonremission.

In the subgroup analyses, we found that female patients with MUNW and MUO had 1.23‐fold and 1.40‐fold increased NR/relapse risk compared with MHNW at 180 days, while male patients with MUNW and MUO were not related to the NR/relapse risk (Figure 2A). Interestingly, male patients with MUNW (90‐day readmission: aOR = 1.19, 95% CI, 1.04–1.36; 30‐day readmission: aOR = 1.18, 95% CI, 1.04, 1.34) and MUO (90‐day readmission: aOR = 1.27, 95% CI, 1.03–1.56; 30‐day readmission: aOR = 1.32, 95% CI, 1.08–1.61) had a significantly higher risk of NR/relapse compared with MHNW. Similarly, female patients with MUNW (90‐day readmission: aOR = 1.11, 95% CI, 0.96–1.30; 30‐day readmission: aOR = 1.09, 95% CI, 0.94–1.25) and MUO (90‐day readmission: aOR = 1.58, 95% CI, 1.27–1.97; 30‐day readmission: aOR = 1.57, 95% CI, 1.28–1.92) had a higher risk of NR/relapse compared with MHNW. Even though the statistical significance was not reached in the female patients with MUNW, the risk of NR/relapse seemed to be higher than in those with MHNW. Therefore, we found that patients with MUNW and MUO had a higher risk of NR/relapse risk at 90 and 30 days compared with MHNW, regardless of gender (Table S5 and S6 in Data S1). We found that patients with MUNW and MUO had a significantly higher risk of severe mortality and disease burden compared with MHNW, regardless of gender (Figure 2C,D). While MHNW female patients were used as the reference group, female patients with MHO had a higher risk of severe mortality (aOR = 1.55, 95% CI, 1.08–2.23) and disease burden (aOR = 1.33, 95% CI, 1.00–1.76). In comparison, male patients with MHO were not related to the risk of severe mortality and disease burden. Therefore, the severe mortality risk and disease burden in the MHO group were different between males and females (Figure 2C,D). Age subgroups demonstrated similar relationships to the total population after adjusting for multiple variables (Table 2, Tables S5 and S6 in Data S1).

### 3.3 Adverse outcomes in patients with CML in further metabolic obesity analysis

The risk of adverse outcomes gradually increased with the number of metabolic risk factors compared to patients without metabolic abnormalities. In addition, obesity exacerbated the negative effects of metabolic abnormalities on adverse outcomes of CML compared to patients with normal weight (Table S7 in Data S1). Meanwhile, similar trends were observed in the sensitivity analyses (Table S8 and S9 in Data S1). We also observed that the risk of adverse outcomes in patients with normal weight only with hyperglycemia and obesity only with hypertension was higher than in normal weight without metabolic abnormalities. Furthermore, patients with obesity only with hyperlipidemia had a higher risk of severe mortality (Table S10 in Data S1). In the sensitivity analyses, we also found that simple hypertension was an independent risk factor for adverse outcomes, irrespective of obesity (Tables S11 and S12 in Data S1).

### 3.4 Severity of illness in patients with CML with metabolically defined obesity

After adjusting for age, sex, total charges, length of stay, admission types, rehab transfer, same‐day events, resident, insurance status, income, location of patient's residence, disposition of patient, and total comorbidities, patients with MUNW (aOR = 1.32; 95% CI, 1.17–1.49), MHO (aOR = 1.36; 95% CI, 1.09–1.70), and MUO (aOR = 1.79; 95% CI, 1.50–2.14) had increased major or extreme loss‐of‐function risk compared with MHNW. Similar trends were also observed in the sensitivity analyses (Table S13 in Data S1).

## 4 DISCUSSION

In this large‐scale national retrospective cohort study, we found that MUNO and MUO were all risk factors for adverse outcomes in patients with CML compared with MHNW. Similar trends with adverse outcomes were also observed on different readmission days. Furthermore, sex differences in the relationship between metabolically defined obesity and NR/relapse of CML were observed. Further analyses demonstrated that patients with a higher number of metabolic risk factors or with dyslipidemia were at higher risk of adverse outcomes, regardless of obesity status. To our knowledge, our study is the first to date to investigate the association between metabolically defined obesity and adverse outcomes in patients with CML.

Obesity is a preventable risk factor for adverse prognosis in cancer patients and is associated with cancer development, relapse, and death. Many mechanisms linking obesity to cancer prognosis are suggested,^19^ such as insulin resistance, metabolic disorders, systemic inflammation, sex hormones pathways, and secretion of a diversity of adipokines. It is likely to play a role in fueling cancer cell growth. For example, patients with obesity with non‐Hodgkin lymphoma, multiple myeloma, and leukemia have a higher risk of death.^7^ Overweight or obesity accounted for 6.5% of all cancer deaths in the United States in 2014, according to the American Cancer Society.^20^ However, we found that MHO, as a protective factor, was not related to adverse health outcomes in patients with CML. Previous studies have also found that higher adiposity is associated with better clinical outcomes and better treatment responses like lung cancer, bladder cancer, and breast cancer.^21^, ^22^ Thus, we discovered the complexity of obesity and its effect on metabolism in patients with CML. Metabolically defined obesity appears to explain this phenomenon, and individuals might benefit from the obesity‐related phenotype. Therefore, it is tempting to speculate that reduced fat depots in the skeletal muscle, liver fat deposition, and subcutaneous adipose tissue expansion in individuals with MHO may account for significant portions of the underlying mechanisms.^23^, ^24^ The metabolic abnormalities contribute more to the adverse prognosis of CML than obesity. Previous studies have also demonstrated that metabolic capability has a greater impact on all‐cause mortality than obesity alone^25^ because metabolic disorders are related to abnormal distribution of body fat, insulin resistance, lipid deposition, the elevation of blood pressure, and inflammation compared to obesity alone.^14^ Nevertheless, there is a lack of consistent results regarding MHO and cancer. Eight prospective cohort studies reported that individuals with MHO are positively associated with cancer incidence, and the effect of obesity is greater than that of metabolic abnormalities.^26^ Although recent studies have reported conflicting results, metabolically defined obesity will provide a more personalized and risk‐stratified approach to obesity treatment, which is precisely the point of our study.

In the subgroup analyses, we investigated whether gender or age affects the association between metabolically defined obesity and adverse outcomes in patients with CML. We found that female patients with MUNW and MUO had increased NR/relapse risk compared with MHNW at 180 days, whereas male patients with MUNW and MUO were not associated with NR/relapse risk. Moreover, patients with MUNW and MUO had a significantly higher risk of NR/relapse at 90 and 30 days compared with MHNW, regardless of gender. Therefore, sex differences in the relationship between metabolically defined obesity and NR/relapse of CML are a long‐term process. The link between obesity and cancer is also thought to involve endogenous levels of estrogen, and then the underlying mechanism may be that estrogen signaling promotes tumor growth by stimulating cellular proliferation, inhibiting apoptosis and inducing angiogenesis.^27^, ^28^ Meanwhile, estrogen may play a crucial role in influencing long‐term results rather than short‐term effects. However, a randomized study with CML from Germany showed that females had a better survival rate of CML than males, without reference to the role of obesity.^29^ Therefore, further studies are needed to determine the mechanism of sex modification in patients with obesity with CML. In addition, as an age‐related neoplasm, the incidence of CML is increasing with the aggravation of population aging in many countries.^30^, ^31^ In our study, obesity seemed to affect adverse outcomes and disease burden in patients aged <55 years, even if it did not reach statistical significance in the MHO group. We found that the proportion of the MHO group was 12.2% and similar to the MHNW group (15.2%) in patients aged <55 years. In comparison, the proportion of the MHO group was 4.9% and far below the MHNW group (45.6%) in patients aged >55 years. Hence, the proportions of the MHO group might drive this difference with age. Furthermore, patients with MHO were determined at baseline. According to previous studies, we found that MHO may shift to other phenotypes over time.^32^ Older people may be more susceptible to weight loss or metabolic abnormalities due to the disease itself or the drugs, which may lead to reverse causation (weight change as a consequence rather than a cause of CML). The patients were followed for only 1 year due to the limitation of the NRD database. Hence, longer cohort studies are needed to explore the mechanism of the age difference. We also found that MUO at all ages was an independent risk factor for adverse outcomes in patients with CML. Future preventive interventions for patients with obesity with CML should be implemented for all ages. Although there are certain limitations that need to be further explored, our study draws further attention to the prognosis of patients with obesity with CML.

According to our knowledge, this is the first study to report the impact of metabolically defined obesity on adverse outcomes in patients with CML. Our study has several unique strengths. Firstly, the 2018 NRD database was designed to study readmission features and hospital‐related outcomes, irrespective of patient insurance status. Secondly, the database involved a large number of inpatients nationwide, which ensured reliable analyses with sufficient sample sizes and ranges. Thirdly, similar results with adverse outcomes in patients with CML were observed in sensitivity analyses using the different readmission days, further supporting our results. Finally, we adjusted for important confounders, such as total comorbidities,^18^ to evaluate adverse outcomes around CML.

Nevertheless, our study has also some limitations. All analyses were derived from the administrative data and ICD‐10‐CM codes. Even if the accuracy of the diagnosis can be assured, there are potential coding errors because of misclassification of diagnosis by physicians and administrative staff. Additionally, the NRD database lacks medication information. Several drugs used to treat type 2 diabetes such as metformin, can inhibit multiple pathways involved in cell growth, which may be associated with anticancer effects.^19^, ^33^ Moreover, BMI in the 2018 NRD database is a categorical variable, not a continuous type. Although we did not use BMI in those of normal weight and above as a continuous, predictor variable in regression model with other metabolic traits, metabolically defined obesity can serve as a model for mechanistic studies regarding obesity and obesity‐associated diseases through the identification of characteristic populations. Finally, the database is year‐based, contains only 1 year of follow‐up data, and excludes death data after discharge.^34^ Hence, we analyzed adverse outcomes in patients with CML based on different readmission days like 30, 90, and 180 days, which enhanced the robustness of our results and minimized the bias due to out‐of‐hospital deaths.

In summary, metabolic abnormalities were related to adverse outcomes in patients with CML, irrespective of obesity status. Obesity would like to aggravate the adverse outcomes in patients with CML, when patients with CML had metabolic abnormalities. Similar findings with adverse outcomes of CML were observed in 30, 90, and 180 days. Future prospective studies should focus on the development and implementation of appropriate interventions in patients with metabolic abnormalities with CML and further elucidate the mechanisms underlying the effects of obesity on their adverse outcomes under different metabolic status, especially in female patients.

## CONFLICT OF INTEREST STATEMENT

The authors declare no competing interests.

## Supporting information


**Figure S1.** Study design, related to methods.

**Data S1.** Supporting information.
