# Supplementary figures and images for: Association of obesity under different metabolic status with adverse outcomes in patients with chronic myeloid leukemia: A retrospective cohort study
Source: J Diabetes. 2023 Apr 28;15(5):436–47. doi: 10.1111/1753-0407.13383 (PMC10172021; doi:10.1111/1753-0407.13383)

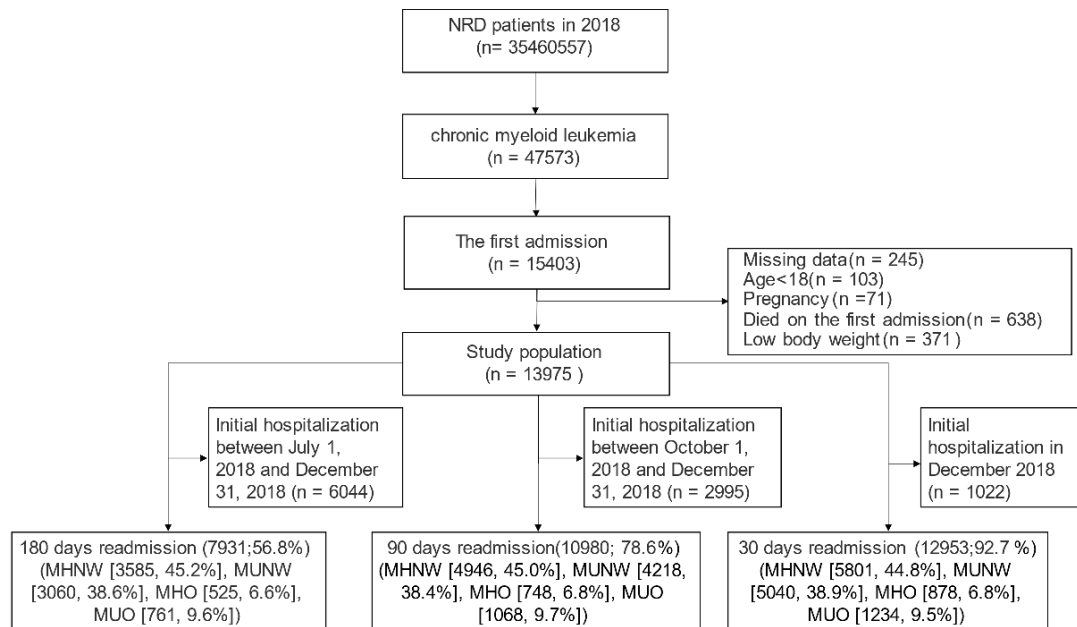

**Supplementary Figure 1. Study design, related to methods.**

Supplement: Supplementary file 1 — Figure S1. Study design, related to methods. [file JDB-15-436-s001.pdf]
