# Supplementary material for: Association of obesity under different metabolic status with adverse outcomes in patients with chronic myeloid leukemia: A retrospective cohort study
Source: J Diabetes. 2023 Apr 28;15(5):436–47. doi: 10.1111/1753-0407.13383 (PMC10172021; doi:10.1111/1753-0407.13383)
Supplement: Supplementary file 2 — Data S1. Supporting information. [file JDB-15-436-s002.docx]

**Supplementary Table 1. Diagnosis codes, used in including and excluding, related to methods.**

| **Diagnoses** | **ICD-10 Codes** |
| --- | --- |
| **Chronic myeloid leukemia** | C9210, C9211, C9212, C9220, C9221, C9222 |
| **Chronic myeloid leukemia, in non-remission (NR)/relapse** | C9210, C9212, C9220, C9222 |
| **Low body weight** | Z681 |
| **Pregnancy** | Z331, Z332, Z333, Z3400, Z3401, Z3402, Z3403, Z3480, Z3481, Z3482, Z3483,  Z3491, Z3492, Z3493, Z36, Z360, Z361, Z362, Z363, Z364, Z365, Z3681, Z3682,  Z3683, Z3684, Z3685, Z3686, Z3687, Z3688, Z3689, Z368A, Z369, Z3A00, Z3A01,  Z3A08, Z3A09, Z3A10, Z3A11, Z3A12, Z3A13, Z3A14, Z3A15, Z3A16, Z3A17, Z3A19,  Z3A18, Z3A20, Z3A21, Z3A22, Z3A23, Z3A24, Z3A25, Z3A26, Z3A27, Z3A28, Z3A29,  Z3A30, Z3A31, Z3A32, Z3A33, Z3A34, Z3A35, Z3A36, Z3A37, Z3A38, Z3A39, Z3A40,  Z3A41, Z3A42, Z3A49, O000, O0000, O0001 |

ICD-10-CM, International Classification of Diseases, Tenth Revision, Clinical Modification.

**Supplementary Table 2. Diagnosis codes, used in classifying patients, related to methods.**

| **Diagnoses** | **ICD-10 Codes** |
| --- | --- |
| **Normal body weight** | Z6820, Z6821, Z6822, Z6823, Z6824 |
| **Overweight** | Z6825, Z6826, Z6827, Z6828, Z6829, E663, DE660A, E669O |
| **Obesity in grade 1** | Z6830, Z6831, Z6832, Z6833, Z6834, DE660B |
| **Obesity in grade 2** | Z6835, Z6836, Z6837, Z6838, Z6839, DE660C |
| **Obesity in grade 3** | Z6841, Z6842, Z6843, Z6844, Z6845, E6601, E662, DE660E, DE660F, DE660G, DE660H |
| **Hyperlipidemia** | **Hypercholesteremia**  E780, E7800, E7801 |
|  | **Hypertriglyceridemia**  E781 |
|  | **Others**  E782, E784, E7849, E785, E783, E7841 |
| **Hypertension** | H35031, H35032, H35033, H35039, G932, I10, I110, I119, I120, I129, I130, I1310, I1311, I132, I150, I151, I152, I158, I159, I160, I161, I169, I674, R030, I973 |
| **Hyperglycemia** | **Prediabetes**  R7301, R7302, R7303 |
|  | **T1DM** |
|  | E108, E109, E1010, E1011, E1021, E1022, E1029, E10311, E10319, E10321, E103213, E103212, E103211, E103219, E10329, E103293, E103292, E103291, E103299, E10331, E103313, E103312, E103311, E103319, E10339, E103393, E103392, E103391, E103399, E10341, E103413, E103412, E103411, E103419, E10349, E103493, E103492, E103491, E103499, E10351, E103513, E103512, E103511, E103519, E103523, E103522, E103521, E103529, E103533, E103532, E103531, E103539, E103543, E103542, E103541, E103549, E103553, E103552, E103551, E103559, E10359, E103593, E103592, E103591, E103599, E1036, E1039, E1037X3, E1037X2, E1037X1, E1037X9, E1040, E1041, E1042, E1044, E1043, E1049, E1051, E1052, E1059, E10610, E10618, E10620, E10621, E10622, E10628, E10630, E10638, E10641, E10649, E1065, E1069 |
|  | **T2DM** |
|  | E1100, E1101, E1111, E1110, E1122, E1121, E1129, E11311, E11319, E11321, E113213, E113212, E113211, E113219, E11329, E113293, E113292, E113291, E113299, E11331, E113313, E113312, E113311, E113319, E11339, E113393, E113392, E113391, E113399, E11341, E113413, E113412, E113411, E113419, E11349, E113493, E113492, E113491, E113499, E11351, E113513, E113512, E113511, E113519, E113523, E113522, E113521, E113529, E113533, E113532, E113531, E113539, E113543, E113542, E113541, E113549, E113553, E113552, E113551, E113559, E11359, E113593, E113592, E113591, E113599, E1136, E1139, E1144, E1143, E1141, E1140, E1142, E1149, E1152, E1151, E1159, E11620, E11621, E11622, E11628, E11610, E11618, E11630, E11638, E1137X3, E1137X2, E1137X1, E1137X9, E11641, E11649, E1165, E1169, E118, E119 |
|  | **Diabetic complication** |
|  | E0800, E0801, E0810, E0811, E0821, E0829, E08311, E08319, E08321, E083211, E083212, E083213, E083219, E08329, E083291, E083292, E083293, E083299, E08331, E083311, E083312, E083313, E083319, E08339, E083391, E083392, E083393, E083399, E08341, E083411, E083412, E083413, E083419, E08349, E083491, E083492, E083493, E083499, E08351, E083511, E083512, E083513, E083519, E083521, E083522, E083523, E083529, E083531, E083532, E083543, E083549, E083551, E083552, E083553, E083559, E08359, E083591, E083592, E083593, E083599, E0836, E0837X1, E0837X2, E0837X3, E0837X9, E0839, E0840, E0841, E0842, E0843, E0844, E0849, E0851, E0852, E0859, E08610, E08618, E08620, E08621, E08622, E08628, E08630, E08638, E08641, E08649, E0865, E0869, E088, E089 |
|  | **Others** |
|  | E1300, E1301, E1310, E1311, E1321, E1322, E1329, E13311, E13319, E13321, E133213, E133212, E133211, E133219, E13329, E133293, E133292, E133291, E133299,E13331, E133313, E133312, E133311, E133319, E13339, E133393, E133392, E133391, E133399, E13341, E133413, E133412, E133411, E133419, E13349, E133493, E133492, E133491, E133499, E13351, E133513, E133512, E133511, E133519, E133523, E133522, E133521, E133529, E133533, E133532, E133531, E133539, E133543, E133542, E133541, E133549, E133553, E133552, E133551, E133559, E13359, E133593, E133592, E133591, E133599, E1340, E1341, E1342, E1343, E1344, E1349, E1336, E1339, E1351, E1352, E1359, E13610, E13618, E13620, E13621, E13622, E13630, E13638, E13641, E13649, E1365, E1369, E1337X3, E1337X2, E1337X1, E1337X9, E138, E139 |
| **Ischemic heart disease** | I248, I249, I2589, I259, Z8249 |
| **Chronic obstructive pulmonary disease (including Chronic bronchitis and emphysema)**  **Neurologic conditions (including dementia, Parkinson's disease and convulsions)** | J410, J411, J418, J42, J430, J431, J432, J438, J439, J440, J441, J449, J470, J471, J479, J8417, J84178, J8489, J849, J982, J983, P250, P258, T797XXA, T797XXD, T797XXS, T8182XA, T8182XD, T8182XS  F0150, F0151, F0280, F0281, F0390, F0391, F1027, F1097, F1327, F1397, F1817, F1827, F1897, F1917, F1927, F1997, G3109, G3183, G2111, G2119, G212, G213, G214, G218, G219, G40001, G40009, G40011, G40019, G40101, G40109, G40111, G40119, G40201, G40209, G40211, G40219, G40301, G40309, G40311, G40319, G40401, G40409, G40411, G40419, G4042, G40501, G40509, G40801, G40802, G40803, G40804, G40811, G40812, G40813, G40814, G40821, G40822, G40823, G40824, G40833, G40834, G4089, G40901, G40909, G40911, G40919, G40A01, G40A09, G40A11, G40A19, G40B01, G40B09, G40B11, G40B19, P90, R5600, R5601, R561, R569 |
| **Chronic kidney disease** | D631, E0922, N181, N182, N183, N1830, N1831, N1832, N184, N185, N186, N189 |
| **Breast cancer** | C50, C500, C5001, C50011, C50012, C50019, C5002, C50021, C50022, C50029, C501, C5011, C50111, C50112, C50119, C5012, C50121, C50122, C50129, C502, C5021, C50211, C50212, C50219, C5022, C50221, C50222, C50229, C503, C5031, C50311, C50312, C50319, C5032, C50321, C50322, C50329, C504, C5041, C50411, C50412, C50419, C5042, C50421, C50422, C50429, C505, C5051, C50511, C50512, C50519, C5052, C50521, C50522, C50529, C506, C5061, C50611, C50612, C50619, C5062, C50621, C50622, C50629, C508, C5081, C50811, C50812, C50819, C5082, C50821, C50822, C50829, C509, C5091, C50911, C50912, C50919, C5092, C50921, C50922, C50929, D05, D050, D0500, D0501, D0502, D051, D0510, D0511, D0512, D058, D0580, D0581, D0582, D059, D0590, D0591, D0592 |
| **Endometrial cancer** | C541, D070 |
| **Cervical cancer** | C530, C531, C538, C539 |
| **Ovarian cancer** | C561, C562, C569 |
| **Esophagus cancer** | C153, C154, C155, C158, C159 |
| **Gastric cancer** | C160, C161, C162, C164, C165, C166, C168, C169 |
| **Liver cancer** | C220, C221, C222, C223, C224, C227, C228, C229 |
| **Pancreatic cancer** | C250, C251, C252, C253, C254, C257, C258, C259 |
| **Colorectal cancer** | C18, C180, C181, C182, C183, C184, C185, C186, C187, C188, C189, C19, C20, C21, C210, C211, C212, C218, D010, D011, D012, D013 |
| **Primary pulmonary carcinoma** | C3410, C3411, C3412, C342, C3430, C3431, C3432, C3480, C3481, C3482, C3400, C3401, C3402, C3490, C3491, C3492 |
| **Prostatic cancer** | C61 |
| **Bladder cancer** | C670, C671, C672, C673, C674, C675, C676, C677, C678, C679, D090 |

ICD-10-CM, International Classification of Diseases, Tenth Revision, Clinical Modification; T1DM, type 1 diabetes mellitus; T2DM, type 2 diabetes mellitus.

**Supplementary Table 3. Baseline characteristics of patients with CML with metabolically defined obesity in 90 days.**

| **Variable** | **Total (n = 10980)** | **MHNW (n = 4946)** | **MUNW (n = 4218)** | **MHO (n = 748)** | **MUO (n = 1068)** | **P value** |
| --- | --- | --- | --- | --- | --- | --- |
| **Age (years) median (IQR)** | 69 (57, 78) | 64 (50, 77) ^a^ | 74 (65, 81) ^b^ | 58 (47, 67) ^c^ | 66 (56, 74) ^a^ | < 0.001 |
| **Age ≥ 55 years, n (%)** | 8448 (76.9) | 3361 (68) ^a^ | 3843 (91.1) ^b^ | 422 (56.4) ^c^ | 822 (77) ^d^ | < 0.001 |
| **Male, n (%)** | 6226 (56.7) | 2773 (56.1) ^a^ | 2501 (59.3) ^b^ | 362 (48.5) ^c^ | 590 (55.2) ^a,b^ | < 0.001 |
| **Total charges ($) median (IQR)** | 39344.0 (21471.0, 76429.0) | 37612.0 (20884.09, 75752.0) ^a^ | 39188.52 (21114.0, 72428.70) ^a^ | 46687.42 (26924.0, 90893.42) ^b^ | 42100.00 (23012.91, 82117.0) ^b^ | < 0.001 |
| **Length of stay (days) median (95% CI )** | 4.00 (6.22, 6.58) | 4.00 (6.37, 6.89) ^a^ | 4.00 (5.58, 5.39) ^a^ | 5.00 (7.33,  8.79) ^b^ | 4.00 (6.37, 8.43) ^c^ | < 0.001 |
| **Non-elective, n (%)** | 9525 (86.8) | 4282 (86.6) ^a^ | 3716 (88.1) ^a^ | 614 (82.1) ^b^ | 913 (85.6) ^a,b^ | < 0.001 |
| **Rehab transfer, n (%)** | 165 (1.5) | 74 (1.5) ^a^ | 70 (1.7) ^a^ | 4 (0.5) ^a^ | 17 (1.6) ^a^ | 0.140 |
| **Same day events, n (%)** | 516 (4.7) | 228 (4.6) ^a^ | 184 (4.4) ^a^ | 42 (5.6) ^a^ | 62 (5.8) ^a^ | 0.140 |
| **Resident, n (%)** | 10250 (93.4) | 4593 (92.9) ^a,b^ | 3957 (93.8) ^a,b^ | 686 (91.7) ^b^ | 1014 (94.9) ^a^ | 0.011 |
| **Insurance status, n (%)** |  |  |  |  |  | < 0.001 |
| Medicare | 7155 (65.2) | 2730 (55.2) ^a^ | 3301(78.3) ^b^ | 375 (50.2) ^a^ | 749 (70.1) ^c^ |  |
| Medicaid | 1011 (9.2) | 646 (13.1) ^a^ | 190 (4.5) ^b^ | 98 (13.1) ^a^ | 77 (7.2) ^c^ |  |
| Private insurance | 2374 (21.6) | 1305 (26.4) ^a^ | 613 (14.5) ^b^ | 235 (31.5) ^c^ | 221 (20.7) ^d^ |  |
| Self-pay | 164 (1.5) | 124 (2.5) ^a^ | 16 (0.4) ^b^ | 13 (1.7) ^a,c^ | 11 (1.0) ^c^ |  |
| No charge | 24 (0.2) | 11 (0.2) ^a^ | 6 (0.1) ^a^ | 4 (0.5) ^a^ | 3 (0.3) ^a^ |  |
| Other | 251 (2.3) | 131 (2.6) ^a^ | 91 (2.2) ^a^ | 22 (2.9) ^a^ | 7 (0.7) ^b^ |  |
| **Median household income by ZIP**  **Code, n (%)** |  |  |  |  |  | < 0.001 |
| $1-$45,999 | 2781 (25.3) | 1254 (25.4) ^a^ | 1042 (24.7) ^a^ | 208 (27.8) ^a^ | 277 (25.9) ^a^ |  |
| $46,000-$58,999 | 3305 (30.1) | 1467 (29.7) ^a,b^ | 1196 (28.4) ^b^ | 253 (33.8) ^a,c^ | 389 (36.4) ^c^ |  |
| $59,000-$78,999 | 2656 (24.2) | 1190 (24.1) ^a^ | 1063 (25.2) ^a^ | 168 (22.4) ^a^ | 235 (22.0) ^a^ |  |
| > $79,000 | 2238 (20.4) | 1035 (20.9) ^a^ | 916 (21.7) ^a^ | 120 (16.0) ^b^ | 167 (15.6) ^b^ |  |
| **Location of patient’s residence, n (%)** |  |  |  |  |  | < 0.001 |
| Large central counties | 2586 (23.6) | 1244 (25.2) ^a^ | 965 (22.9) ^a^ | 132 (17.7) ^b^ | 245 (23.0) ^a^ |  |
| Large fringe counties | 3029 (27.6) | 1355 (27.4) ^a^ | 1228 (29.1) ^a^ | 202 (27.0) ^a,b^ | 244 (22.9) ^b^ |  |
| Medium metro counties | 2350 (21.4) | 990 (20.0) ^a^ | 917 (21.7) ^a,b^ | 182 (24.4) ^b^ | 261 (24.5) ^b^ |  |
| Small metro counties | 1098 (10.0) | 508 (10.3) ^a,b^ | 383 (9.1) ^b^ | 78 (10.4) ^a,b^ | 129 (12.1) ^a^ |  |
| Micropolitan counties | 1145 (10.4) | 536 (10.8) ^a^ | 421 (10.0) ^a^ | 81 (10.8) ^a^ | 107 (10.0) ^a^ |  |
| Not metro/micropolitan counties | 771 (7.0) | 313 (6.3) ^a^ | 305 (7.2) ^a,b^ | 72 (9.6) ^b^ | 81 (7.6) ^a,b^ |  |
| **Disposition of patient, n (%)** |  |  |  |  |  | < 0.001 |
| Routine | 6877 (62.6) | 3321 (67.1) ^a^ | 2443 (57.9) ^b^ | 507 (67.8) ^a^ | 606 (56.7) ^b^ |  |
| Transfer to short-term hospital | 123 (1.1) | 48 (1.0) ^a^ | 42 (1.0) ^a^ | 19 (2.5) ^b^ | 14 (1.3) ^a,b^ |  |
| Transfer Other | 1717 (15.6) | 655 (13.2) ^a^ | 790 (18.7) ^b^ | 82 (11.0) ^a^ | 190 (17.8) ^b^ |  |
| Home Health Care | 2150 (19.6) | 849 (17.2) ^a^ | 916 (21.7) ^b,c^ | 133 (17.8) ^a,c^ | 252 (23.6) ^b^ |  |
| Against Medical Advice | 109 (1.0) | 69 (1.4) ^a^ | 27 (0.6) ^b^ | 7 (0.9) ^a,b^ | 6 (0.6) ^a,b^ |  |
| Discharge alive, destination unknown | 4 (0.0) | 4 (0.1) ^a^ | 0 (0.0) ^a^ | 0 (0.0) ^a^ | 0 (0.0) ^a^ |  |
| [**Hyperglycemia**](javascript:;)**, n (%)** | 3637 (33.1) | 307 (6.2) ^a^ | 2440 (57.8) ^b^ | 96 (12.8) ^c^ | 794 (74.3) ^d^ | < 0.001 |
| **Hypertension, n (%)** | 7513 (68.4) | 2039 (41.2) ^a^ | 4086 (96.9) ^b^ | 367 (49.1) ^c^ | 1021 (95.6) ^b^ | < 0.001 |
| **Dyslipidemia, n (%)** | 4491 (40.9) | 382 (7.7) ^a^ | 3300 (78.2) ^b^ | 38 (5.1) ^a^ | 771 (72.2) ^c^ | < 0.001 |
| **Major or extreme loss of function, n (%)** | 6537 (59.5) | 2641 (53.4) ^a^ | 2667 (63.2) ^b^ | 465 (62.2) ^b^ | 764 (71.5) ^c^ | < 0.001 |
| **Risk of severe mortality, n (%)** | 4781 (43.5) | 1817 (36.7) ^a^ | 2144 (50.8) ^b^ | 288 (38.5) ^a^ | 532 (49.8) ^b^ | < 0.001 |
| **Comorbidities** |  |  |  |  |  |  |
| Ischemic heart disease, n (%) | 178 (1.6) | 40 (0.8) ^a^ | 101 (2.4) ^b^ | 15 (2.0) ^b^ | 22 (2.1) ^b^ | < 0.001 |
| Chronic obstructive pulmonary disease, n (%) | 168 (1.5) | 63 (1.3) ^a,b,c^ | 80 (1.9) ^c^ | 2 (0.3) ^b^ | 23 (2.2) ^a,c^ | 0.001 |
| Neurologic condition, n (%) | 464 (4.2) | 218 (4.4) ^a^ | 180 (4.3) ^a^ | 26 (3.5) ^a^ | 40 (3.7) ^a^ | 0.560 |
| Chronic kidney disease, n (%) | 483 (4.4) | 117 (2.4) ^a^ | 283 (6.7) ^b^ | 10 (1.3) ^a^ | 73 (6.8) ^b^ | < 0.001 |
| Female reproductive malignancy, n (%) | 62 (0.6) | 23 (0.5) ^a^ | 27 (0.6) ^a^ | 3 (0.4) ^a^ | 9 (0.8) ^a^ | 0.372 |
| Digestive system malignancy, n (%) | 87 (0.8) | 41 (0.8) ^a^ | 37 (0.9) ^a^ | 0 (0.0) ^a^ | 9 (0.8) ^a^ | 0.090 |
| Primary Pulmonary Carcinoma, n (%) | 95 (0.9) | 41 (0.8) ^a^ | 34 (0.8) ^a^ | 9 (1.2) ^a^ | 11 (1.0) ^a^ | 0.664 |
| Malignant tumor of the urinary system, n (%) | 151 (1.4) | 69 (1.4) ^a^ | 55 (1.3) ^a^ | 13 (1.7) ^a^ | 14 (1.3) ^a^ | 0.818 |
| **Total comorbidities, n (%)** |  |  |  |  |  | < 0.001 |
| **< 4** | 9622 (87.6) | 4438 (89.7) ^a^ | 3586 (85.0) ^b^ | 693 (92.6) ^a^ | 905 (84.7) ^b^ |  |

The small letters (e. g. a, b, c, d, etc.) in this table refer to comparisons between groups. There is no statistical difference between groups with the same small letters.

CML, chronic myeloid leukemia; MHNW, metabolically healthy normal weight; MUNW, metabolically unhealthy normal weight; MHO, metabolically healthy obesity; MUO, metabolically unhealthy obesity.

**Supplementary Table 4. Baseline characteristics of patients with CML with** **metabolically defined obesity in 30 days.**

| **Variable** | **Total (n = 12953)** | **MHNW (n = 5801)** | **MUNW (n = 5040)** | **MHO (n = 878)** | **MUO (n = 1234)** | **P value** |
| --- | --- | --- | --- | --- | --- | --- |
| **Age (years) median (IQR)** | 69 (57, 78) | 64 (50, 77) ^a^ | 74 (65, 81) ^b^ | 58 (48, 67) ^c^ | 67 (56, 74) ^a^ | < 0.001 |
| **Age ≥ 55 years, n (%)** | 10003 (77.2) | 3936 (67.9) ^a^ | 4616 (91.6) ^b^ | 501 (57.1) ^c^ | 950 (77.0) ^d^ | < 0.001 |
| **Male, n (%)** | 7302 (56.4) | 3243 (55.9) ^a^ | 2968 (58.9) ^b^ | 424 (48.3) ^c^ | 667 (54.1) ^a,c^ | < 0.001 |
| **Total charges ($) median (IQR)** | 39382.63 (21558.0, 77297.37) | 37208.0 (20584.14, 75474.81) ^a^ | 39830.0 (21585.68, 73903.0) ^a^ | 46478.34 (25977.17, 90820.0) ^b^ | 43028.0 (22938.35, 84800.0) ^b^ | < 0.001 |
| **Length of stay (days) median (****95% CI )** | 4.00 (6.20, 6.52) | 4.00 (6.30, 6.78) ^a^ | 4.00 (5.46, 5.80) ^a^ | 5.00 (7.21, 8.50) ^b^ | 4.00 (6.47,8.30) ^c^ | < 0.001 |
| **Non-elective, n (%)** | 11240 (86.8) | 5043 (86.9) ^a,b^ | 4433 (88.0) ^b^ | 720 (82.0) ^c^ | 1044 (84.6) ^a,c^ | < 0.001 |
| **Rehab transfer, n (%)** | 194 (1.5) | 77 (1.3) ^a^ | 90 (1.8) ^a^ | 8 (0.9) ^a^ | 19 (1.5) ^a^ | 0.110 |
| **Same day events, n (%)** | 606 (4.7) | 256 (4.4) ^a^ | 230 (4.6) ^a^ | 53 (6.0) ^a^ | 67 (5.4) ^a^ | 0.100 |
| **Resident, n (%)** | 12101 (93.4) | 539 (92.9) ^a,b,c^ | 4740 (94.1) ^c^ | 800 (91.1) ^b^ | 1170 (94.8) ^a,c^ | 0.001 |
| **Insurance status, n (%)** |  |  |  |  |  | < 0.001 |
| Medicare | 8447 (65.2) | 3216 (55.4) ^a^ | 3953 (78.4) ^b^ | 415 (47.2) ^c^ | 863 (69.9) ^d^ |  |
| Medicaid | 1199 (9.3) | 759 (13.1) ^a^ | 224 (4.4) ^b^ | 120 (13.7) ^a^ | 96 (7.8) ^c^ |  |
| Private insurance | 2800 (21.6) | 1518 (26.2) ^a^ | 733 (14.5) ^b^ | 298 (33.9) ^c^ | 251 (20.3) ^d^ |  |
| Self-pay | 185 (1.4) | 143 (2.5) ^a^ | 16 (0.3) ^b^ | 15 (1.7) ^a,c^ | 11 (0.9) ^c^ |  |
| No charge | 29 (0.2) | 16 (0.3) ^a^ | 6 (0.1) ^a^ | 4 (0.5) ^a^ | 3 (0.2) ^a^ |  |
| Other | 294 (2.3) | 149 (2.6) ^a^ | 108 (2.1) ^a^ | 27 (3.1) ^a^ | 10 (0.8) ^b^ |  |
| **Median household income by ZIP**  **Code, n (%)** |  |  |  |  |  | < 0.001 |
| $1-$45,999 | 3298 (25.5) | 1479 (25.5) ^a^ | 1264 (25.1) ^a^ | 237 (27.0) ^a^ | 318 (25.8) ^a^ |  |
| $46,000-$58,999 | 3859 (29.8) | 1696 (29.2) ^a,b^ | 1415 (28.1) ^b^ | 295 (33.6) ^a,c^ | 453 (36.7) ^c^ |  |
| $59,000-$78,999 | 3175 (24.5) | 1413 (24.4) ^a^ | 1283 (25.5) ^a^ | 205 (23.3) ^a^ | 274 (22.2) ^a^ |  |
| > $79,000 | 2620 (20.2) | 1213 (20.9) ^a^ | 1078 (21.4) ^a^ | 141 (16.1) ^b^ | 188 (15.2) ^b^ |  |
| **Location of patient’s residence, n (%)** |  |  |  |  |  | < 0.001 |
| Large central counties | 3082 (23.8) | 1429 (24.6) ^a^ | 1195 (23.7) ^a^ | 169 (19.2) ^b^ | 289 (23.4) ^a,b^ |  |
| Large fringe counties | 3570 (27.6) | 1613 (27.8) ^a^ | 1449 (28.8) ^a^ | 233 (26.5) ^a,b^ | 275 (22.3) ^b^ |  |
| Medium metro counties | 2785 (21.5) | 1117 (20.3) ^a^ | 1087 (21.6) ^a,b^ | 216 (24.6) ^b^ | 305 (24.7) ^b^ |  |
| Small metro counties | 1278 (9.9) | 591 (10.2) ^a,b^ | 454 (9.0) ^b^ | 85 (9.7) ^a,b^ | 148 (12.0) ^a^ |  |
| Micropolitan counties | 1317 (10.2) | 613 (10.6) ^a^ | 495 (9.8) ^a^ | 91 (10.4) ^a^ | 118 (9.6) ^a^ |  |
| Not metro/micropolitan counties | 920 (7.1) | 379 (6.5) ^a^ | 359 (7.1) ^a,b^ | 84 (9.6) ^b^ | 98 (7.9) ^a,b^ |  |
| **Disposition of patient, n (%)** |  |  |  |  |  | < 0.001 |
| Routine | 8089 (62.4) | 3896 (67.1) ^a^ | 2882 (57.2) ^b^ | 605 (68.8) ^a^ | 706 (57.3) ^b^ |  |
| Transfer to short-term hospital | 140 (1.1) | 55 (0.9) ^a^ | 50 (1.0) ^a^ | 19 (2.2) ^b^ | 16 (1.3) ^a,b^ |  |
| Transfer Other | 2056 (15.9) | 788 (13.6) ^a^ | 960 (19.1) ^b^ | 93 (10.6) ^a^ | 215 (17.4) ^b^ |  |
| Home Health Care | 2545 (19.6) | 981 (16.9) ^a^ | 1119 (22.2) ^b^ | 155 (17.6) ^a^ | 290 (23.5) ^b^ |  |
| Against Medical Advice | 117 (0.9) | 76 (1.3) ^a^ | 28 (0.6) ^b^ | 7 (0.8) ^a,b^ | 6 (0.5) ^a,b^ |  |
| Discharge alive, destination unknown | 6 (0.0) | 6 (0.1) ^a^ | 0 (0.0) ^a^ | 0 (0.0) ^a^ | 0 (0.0) ^a^ |  |
| [**Hyperglycemia**](javascript:;)**, n (%)** | 4276 (33.0) | 352 (6.1) ^a^ | 2903 (57.6) ^b^ | 110 (12.5) ^c^ | 911 (73.8) ^d^ | < 0.001 |
| **Hypertension, n (%)** | 8877 (68.5) | 2380 (41.0) ^a^ | 4891 (97.0) ^b^ | 422 (48.1) ^c^ | 1184 (95.9) ^b^ | < 0.001 |
| **Dyslipidemia, n (%)** | 5337 (41.2) | 456 (7.9) ^a^ | 3947 (78.3) ^b^ | 50 (5.7) ^a^ | 884 (71.6) ^c^ | < 0.001 |
| **Major or extreme loss of function, n (%)** | 7670 (59.2) | 3071 (52.9) ^a^ | 3176 (63.0) ^b^ | 546 (62.2) ^b^ | 877 (71.1) ^c^ | < 0.001 |
| **Risk of severe mortality, n (%)** | 5610 (43.3) | 2114 (36.4) ^a^ | 2563(50.9) ^b^ | 326 (37.1) ^a^ | 607 (49.2) ^b^ | < 0.001 |
| **Comorbidities** |  |  |  |  |  |  |
| Ischemic heart disease, n (%) | 218 (1.7) | 51 (0.9) ^a^ | 127 (2.5) ^b^ | 15 (1.7) ^a,b^ | 25 (2.0) ^b^ | < 0.001 |
| Chronic obstructive pulmonary disease, n (%) | 203 (1.6) | 83 (1.4) ^a^ | 90 (1.8) ^a^ | 2 (0.2) ^b^ | 28 (2.3) ^a^ | 0.001 |
| Neurologic condition, n (%) | 535 (4.1) | 233 (4.0) ^a^ | 217 (4.3) ^a^ | 31 (3.5) ^a^ | 54 (4.4) ^a^ | 0.667 |
| Chronic kidney disease, n (%) | 599 (4.6) | 145 (2.5) ^a^ | 363 (7.2) ^b^ | 10 (1.1) ^a^ | 81 (6.6) ^b^ | < 0.001 |
| Female reproductive malignancy, n (%) | 75 (0.6) | 31 (0.5) ^a^ | 31 (0.6) ^a^ | 3 (0.3) ^a^ | 10 (0.8) ^a^ | 0.509 |
| Digestive system malignancy, n (%) | 112 (0.9) | 50 (0.9) ^a^ | 52 (1.0) ^a^ | 0 (0.0) ^b^ | 10 (0.8) ^a^ | 0.025 |
| Primary Pulmonary Carcinoma, n (%) | 115 (0.9) | 44 (0.8) ^a^ | 46 (0.9) ^a^ | 11(1.3) ^a^ | 14 (1.1) ^a^ | 0.345 |
| Malignant tumor of the urinary system, n (%) | 174 (1.3) | 84 (1.4) ^a^ | 61 (1.2) ^a^ | 13 (1.5) ^a^ | 16 (1.3) ^a^ | 0.729 |
| **Total comorbidities, n (%)** |  |  |  |  |  | < 0.001 |
| < 4 | 11320 (87.4) | 5204 (89.7) ^a^ | 4260 (84.5) ^b^ | 817 (93.1) ^c^ | 1039 (84.2) ^b^ |  |

The small letters (e. g. a, b, c, d, etc.) in this table refer to comparisons between groups. There is no statistical difference between groups with the same small letters.

CML, chronic myeloid leukemia; MHNW, metabolically healthy normal weight; MUNW, metabolically unhealthy normal weight; MHO, metabolically healthy obesity; MUO, metabolically unhealthy obesity.

**Supplementary Table 5. Association of metabolically defined obesity with multiple adverse outcomes and disease burden in patients with CML in 90 days.**

|  | **NR/Relapse** | **Severe mortality risk** | **Disease burden** |
| --- | --- | --- | --- |
| **Variable** | **aOR (95% CI) P value** | **aOR (95% CI) P value** | **aOR (95% CI) P value** |
| Total |  |  |  |
| MHNW | 1 (Reference) | 1 (Reference) | 1 (Reference) |
| MUNW | 1.16 (1.05,1.28) 0.004 | 1.34 (1.19,1.50) < 0.001 | 1.21 (1.10,1.34) < 0.001 |
| MHO | 1.16 (0.96,1.38) 0.120 | 1.09 (0.86,1.37) 0.490 | 1.15 (0.97,1,37) 0.119 |
| MUO | 1.42 (1.22,1.65) < 0.001 | 1.67 (1.41,1.98) < 0.001 | 1.48 (1.29,1.71) <0.001 |
| Male |  |  |  |
| MHNW | 1 (Reference) | 1 (Reference) | 1 (Reference) |
| MUNW | 1.19 (1.04,1.36) 0.014 | 1.36 (1.16,1.59) < 0.001 | 1.27(1.12,1.45) < 0.001 |
| MHO | 1.18 (0.90,1.53) 0.233 | 1.06 (0.75,1.49) 0.751 | 1.23 (0.96,1.59) 0.100 |
| MUO | 1.27 (1.03,1.56) 0.026 | 1.59 (1.26,2.00) < 0.001 | 1.34 (1.10,1.63) 0.004 |
| Female |  |  |  |
| MHNW | 1 (Reference) | 1 (Reference) | 1 (Reference) |
| MUNW | 1.11(0.96,1.30) 0.172 | 1.32 (1.10,1.59) 0.003 | 1.13 (0.98,1.31) 0.097 |
| MHO | 1.09(0.85,1.40) 0.512 | 1.09 (0.78,1.51) 0.612 | 1.05 (0.82,1.33) 0.724 |
| MUO | 1.58(1.27,1.97) < 0.001 | 1.75 (1.36,2.25) < 0.001 | 1.64 (1.33,2.02) < 0.001 |
| Age < 55 years |  |  |  |
| MHNW | 1 (Reference) | 1 (Reference) | 1 (Reference) |
| MUNW | 1.34 (1.03,1.75) 0.032 | 1.58 (1.08,2.32) 0.018 | 1.41 (1.09,1.82) 0.009 |
| MHO | 1.25 (0.94,1.67) 0.123 | 1.79 (1.21,2.66) 0.004 | 1.30 (0.99,1.71) 0.062 |
| MUO | 1.35 (0.99,1.85) 0.060 | 1.81 (1.19,2.77) 0.006 | 1.37 (1.01,1.85) 0.042 |
| Age ≥ 55 years |  |  |  |
| MHNW | 1 (Reference) | 1 (Reference) | 1 (Reference) |
| MUNW | 1.13 (1.01,1.26) 0.029 | 1.29 (1.14,1.46) < 0.001 | 1.18 (1.06,1.31) 0.002 |
| MHO | 1.06 (0.83,1.35) 0.647 | 0.83 (0.61,1.12) 0.217 | 1.02 (0.81,1.29) 0.864 |
| MUO | 1.40 (1.18,1.66) < 0.001 | 1.62 (1.34,1.96) < 0.001 | 1.46 (1.24,1.72) < 0.001 |

After adjusting for age, sex, total charges, length of stay, admission types, rehab transfer, same day events, resident, insurance status, income, location of patient’s residence, disposition of patient and total comorbidities.

CML, chronic myeloid leukemia; MHNW, metabolically healthy normal weight; MUNW, metabolically unhealthy normal weight; MHO, metabolically healthy obesity; MUO, metabolically unhealthy obesity; NR, non-remission; aOR, adjusted odds ratio; CI, [confidence](javascript:;) [interval](javascript:;).

**Supplementary Table 6. Association of metabolically defined obesity with** **adverse outcomes and disease burden in patients with CML in 30 days.**

|  | **NR/Relapse** | **Severe mortality risk** | **Disease burden** |
| --- | --- | --- | --- |
| **Variable** | **aOR (95% CI) P value** | **aOR (95% CI) P value** | **aOR (95% CI) P value** |
| Total |  |  |  |
| MHNW | 1 (Reference) | 1 (Reference) | 1 (Reference) |
| MUNW | 1.14 (1.04,1.26) 0.006 | 1.33 (1.19,1.49) < 0.001 | 1.19 (1.09,1.31) < 0.001 |
| MHO | 1.14 (0.96,1.36) 0.127 | 1.16 (0.93,1.45) 0.195 | 1.16 (0.99,1.37) 0.070 |
| MUO | 1.45 (1.26,1.67) < 0.001 | 1.68 (1.43,1.98) < 0.001 | 1.50 (1.31,1.71) <0.001 |
| Male |  |  |  |
| MHNW | 1 (Reference) | 1 (Reference) | 1 (Reference) |
| MUNW | 1.18 (1.04,1.34) 0.012 | 1.34 (1.16,1.55) < 0.001 | 1.26 (1.12,1.43) < 0.001 |
| MHO | 1.16 (0.90,1.49) 0.264 | 1.18 (0.86,1.63) 0.297 | 1.26 (1.00,1.60) 0.054 |
| MUO | 1.32 (1.08,1.61) 0.007 | 1.57 (1.26,1.97) < 0.001 | 1.36 (1.13,1.65) 0.001 |
| Female |  |  |  |
| MHNW | 1 (Reference) | 1 (Reference) | 1 (Reference) |
| MUNW | 1.09 (0.94,1.25) 0.270 | 1.32 (1.11,1.56) 0.002 | 1.09 (0.95,1.25) 0.211 |
| MHO | 1.06 (0.83,1.34) 0.645 | 1.09 (0.80,1.50) 0.582 | 1.02 (0.81,1.29) 0.854 |
| MUO | 1.57 (1.28,1.92) < 0.001 | 1.77 (1.40,2.25) < 0.001 | 1.62 (1.33,1.97) <0.001 |
| Age < 55 years |  |  |  |
| MHNW | 1 (Reference) | 1 (Reference) | 1 (Reference) |
| MUNW | 1.30 (1.01,1.69) 0.045 | 1.60 (1.10,2.31) 0.013 | 1.39 (1.09,1.77) 0.009 |
| MHO | 1.27 (0.97,1.66) 0.085 | 1.88 (1.28,2.77) 0.001 | 1.32 (1.02,1.71) 0.036 |
| MUO | 1.36 (1.01,1.84) 0.043 | 1.92 (1.28,2.88) 0.02 | 1.35 (1.01,1.80) 0.042 |
| Age ≥ 55 years |  |  |  |
| MHNW | 1 (Reference) | 1 (Reference) | 1 (Reference) |
| MUNW | 1.11 (1.00,1.24) 0.041 | 1.28 (1.14,1.44) < 0.001 | 1.16 (1.05,1.28) 0.004 |
| MHO | 1.01 (0.80,1.27) 0.916 | 0.90 (0.68,1.20) 0.476 | 1.02 (0.82,1.26) 0.886 |
| MUO | 1.43 (1.22,1.69) < 0.001 | 1.61 (1.34,1.92) < 0.001 | 1.49 (1.27,1.74) <0.001 |

After adjusting for age, sex, total charges, length of stay, admission types, rehab transfer, same day events, resident, insurance status, income, location of patient’s residence, disposition of patient and total comorbidities.

CML, chronic myeloid leukemia; MHNW, metabolically healthy normal weight; MUNW, metabolically unhealthy normal weight; MHO, metabolically healthy obesity; MUO, metabolically unhealthy obesity; NR, non-remission; aOR, adjusted odds ratio; CI, [confidence](javascript:;) [interval](javascript:;).

**Supplementary Table 7. Association of the number of metabolic risk factors with the risk of adverse outcomes and disease burden in patients with CML in 180 days.**

|  | **NR/Relapse** | **Severe mortality risk** | **Disease burden** |
| --- | --- | --- | --- |
| **Variable** | **aOR (95% CI) P value** | **aOR (95% CI) P value** | **aOR (95% CI) P value** |
| Normal weight, no risk | 1 (Reference) | 1 (Reference) | 1 (Reference) |
| Normal weight, 1 risk | 1.18 (1.01,1.39) 0.042 | 1.17 (0.96,1.43) 0.119 | 1.20 (1.03,1.39) 0.023 |
| Normal weight, 2 risks | 1.22 (1.04,1.44) 0.016 | 1.32 (1.09,1.62) 0.006 | 1.26 (1.08,1.47) 0.004 |
| Normal weight, 3 risks | 1.50 (1.25,1.82) < 0.001 | 1.93 (1.55,2.40) < 0.001 | 1.73 (1.44,2.07) < 0.001 |
| Obesity, no risk | 1.07 (0.74,1.54) 0.713 | 0.59 (0.32,1.07) 0.079 | 1.07 (0.75,1.52) 0.723 |
| Obesity, 1 risk | 1.40 (1.08,1.82) 0.012 | 1.57 (1.15,2.16) 0.005 | 1.50 (1.17,1.93) 0.002 |
| Obesity, 2 risks | 1.28 (1.00,1.63) 0.048 | 1.80 (1.37,2.37) < 0.001 | 1.41 (1.12,1.78) 0.004 |
| Obesity, 3 risks | 1.75 (1.34,2.30) < 0.001 | 1.81 (1.32,2.47) < 0.001 | 1.94 (1.50,2.52) < 0.001 |

After adjusting for age, sex, total charges, length of stay, admission types, rehab transfer, same day events, resident, insurance status, income, location of patient’s residence, disposition of patient and total comorbidities.

CML, chronic myeloid leukemia; MHNW, metabolically healthy normal weight; MUNW, metabolically unhealthy normal weight; MHO, metabolically healthy obesity; MUO, metabolically unhealthy obesity; NR, non-remission; aOR, adjusted odds ratio; CI, [confidence](javascript:;) [interval](javascript:;).

**Supplementary Table 8. Association of the number of metabolic risk factors with the risk of adverse outcomes and disease burden in patients with CML in 90 days.**

|  | **NR/Relapse** | **Severe mortality risk** | **Disease burden** |
| --- | --- | --- | --- |
| **Variable** | **aOR (95% CI) P value** | **aOR (95% CI) P value** | **aOR (95% CI) P value** |
| Normal weight, no risk | 1 (Reference) | 1 (Reference) | 1 (Reference) |
| Normal weight, 1 risk | 1.20 (1.04,1.39) 0.010 | 1.26(1.05,1.51) 0.011 | 1.23 (1.08,1.41) 0.002 |
| Normal weight, 2 risks | 1.20 (1.04,1.39) 0.014 | 1.39(1.17,1.66) < 0.001 | 1.26 (1.10,1.44) 0.001 |
| Normal weight, 3 risks | 1.53 (1.30,1.81) < 0.001 | 1.93(1.59,2.35) < 0.001 | 1.68 (1.43,1.96) < 0.001 |
| Obesity, no risk | 1.16 (0.85,1.59) 0.351 | 0.88(0.55,1.41) 0.597 | 1.15 (0.85,1.55) 0.377 |
| Obesity, 1 risk | 1.34 (1.06,1.68) 0.013 | 1.42(1.07,1.89) 0.017 | 1.36 (1.09,1.69) 0.006 |
| Obesity, 2 risks | 1.42 (1.15,1.75) 0.001 | 1.80(1.41,2.30) < 0.001 | 1.49 (1.22,1.82) < 0.001 |
| Obesity, 3 risks | 1.84 (1.46,2.31) < 0.001 | 2.12(1.63,2.77) < 0.001 | 1.98 (1.59,2.46) < 0.001 |

After adjusting for age, sex, total charges, length of stay, admission types, rehab transfer, same day events, resident, insurance status, income, location of patient’s residence, disposition of patient and total comorbidities.

CML, chronic myeloid leukemia; MHNW, metabolically healthy normal weight; MUNW, metabolically unhealthy normal weight; MHO, metabolically healthy obesity; MUO, metabolically unhealthy obesity; NR, non-remission; aOR, adjusted odds ratio; CI, [confidence](javascript:;) [interval](javascript:;).

**Supplementary Table 9. Association of the number of metabolic risk factors with the risk of adverse outcomes and disease burden in patients with CML in 30 days.**

|  | **NR/Relapse** | **Severe mortality risk** | **Disease burden** |
| --- | --- | --- | --- |
| **Variable** | **aOR (95% CI) P value** | **aOR (95% CI) P value** | **aOR (95% CI) P value** |
| Normal weight, no risk | 1 (Reference) | 1 (Reference) | 1 (Reference) |
| Normal weight, 1 risk | 1.23 (1.08,1.41) 0.003 | 1.27 (1.07,1.51) 0.007 | 1.25 (1.10,1.43) 0.001 |
| Normal weight, 2 risks | 1.19 (1.04,1.37) 0.012 | 1.39 (1.18,1.65) < 0.001 | 1.25 (1.10,1.43) 0.001 |
| Normal weight, 3 risks | 1.55 (1.32,1.81) < 0.001 | 1.92 (1.59,2.32) < 0.001 | 1.66 (1.43,1.92) <0.001 |
| Obesity, no risk | 1.11 (0.82,1.50) 0.490 | 0.85 (0.53,1.34) 0.479 | 1.08 (0.81,1.45) 0.588 |
| Obesity, 1 risk | 1.37 (1.10,1.70) 0.005 | 1.57 (1.20,2.06) 0.001 | 1.44 (1.17,1.77) <0.001 |
| Obesity, 2 risks | 1.53 (1.26,1.86) < 0.001 | 1.90 (1.50,2.40) < 0.001 | 1.57 (1.30,1.89) <0.001 |
| Obesity, 3 risks | 1.81 (1.46,2.25) < 0.001 | 2.05 (1.59,2.65) < 0.001 | 1.94 (1.57,2.39) <0.001 |

After adjusting for age, sex, total charges, length of stay, admission types, rehab transfer, same day events, resident, insurance status, income, location of patient’s residence, disposition of patient and total comorbidities.

CML, chronic myeloid leukemia; MHNW, metabolically healthy normal weight; MUNW, metabolically unhealthy normal weight; MHO, metabolically healthy obesity; MUO, metabolically unhealthy obesity; NR, non-remission; aOR, adjusted odds ratio; CI, [confidence](javascript:;) [interval](javascript:;).

**Supplementary Table 10. Association of** **specific metabolic risk factors with the risk of adverse outcomes and disease burden in patients with CML in 180 days.**

|  | **NR/Relapse** | **Severe mortality risk** | **Disease burden** |
| --- | --- | --- | --- |
| Variable | **aOR (95% CI) P value** | **aOR (95% CI) P value** | **aOR (95% CI) P value** |
| Normal weight, no risk | 1 (Reference) | 1 (Reference) | 1 (Reference) |
| Normal weight, hyperglycemia | 1.95 (1.43,2.67) < 0.001 | 2.26 (1.57,3.24) < 0.001 | 1.95 (1.44,2.66) < 0.001 |
| Normal weight, hypertension | 1.11 (0.93,1.32) 0.239 | 1.13 (0.91,1.41) 0.254 | 1.15 (0.97,1.36) 0.105 |
| Normal weight, hyperlipidemia | 0.76 (0.55,1.06) 0.110 | 0.67 (0.44,1.03) 0.066 | 0.75 (0.55,1.03) 0.074 |
| Obesity, no risk | 1.10 (0.76,1.59) 0.619 | 0.60 (0.33,1.10) 0.099 | 1.08 (0.76,1.55) 0.660 |
| Obesity, hyperglycemia | 1.05 (0.59,1.86) 0.863 | 1.78 (0.96,3.33) 0.069 | 1.33 (0.78,2.28) 0.289 |
| Obesity, hypertension | 1.69 (1.25,2.28) 0.001 | 1.54 (1.05,2.24) 0.026 | 1.55 (1.16,2.08) 0.003 |
| Obesity, hyperlipidemia | 0.49 (0.17,1.39) 0.180 | 2.43 (1.04,5.67) 0.041 | 1.99 (0.92,4.28) 0.079 |

After adjusting for age, sex, total charges, length of stay, admission types, rehab transfer, same day events, resident, insurance status, income, location of patient’s residence, disposition of patient and total comorbidities.

CML, chronic myeloid leukemia; MHNW, metabolically healthy normal weight; MUNW, metabolically unhealthy normal weight; MHO, metabolically healthy obesity; MUO, metabolically unhealthy obesity; NR, non-remission; aOR, adjusted odds ratio; CI, [confidence](javascript:;) [interval](javascript:;).

**Supplementary Table 11. Association of specific metabolic risk factors with the risk of adverse outcomes and disease burden in patients with CML in 90 days.**

|  | **NR/Relapse** | **Severe mortality risk** | **Disease burden** |
| --- | --- | --- | --- |
| **Variable** | **aOR (95% CI) P value** | **aOR (95% CI) P value** | **aOR (95% CI) P value** |
| Normal weight, no risk | 1 (Reference) | 1 (Reference) | 1 (Reference) |
| Normal weight, hyperglycemia | 1.65 (1.26,2.16) < 0.001 | 2.01 (1.46,2.76) < 0.001 | 1.72 (1.32,2.23) < 0.001 |
| Normal weight, hypertension | 1.20 (1.03,1.40) 0.022 | 1.21 (1.00,1.47) 0.057 | 1.22 (1.05,1.41) 0.008 |
| Normal weight, hyperlipidemia | 0.77 (0.57,1.04) 0.090 | 0.93 (0.65,1.32) 0.688 | 0.87 (0.66,1.14) 0.319 |
| Obesity, no risk | 1.22 (0.89,1.67) 0.224 | 0.93 (0.58,1.49) 0.757 | 1.19 (0.88,1.62) 0.259 |
| Obesity, hyperglycemia | 0.89 (0.53,1.50) 0.665 | 1.46 (0.81,2.63) 0.209 | 1.05 (0.65,1.70) 0.838 |
| Obesity, hypertension | 1.54 (1.19,1.98) 0.001 | 1.33 (0.96,1.86) 0.090 | 1.39 (1.08,1.78) 0.010 |
| Obesity, hyperlipidemia | 0.73 (0.30,1.81) 0.496 | 2.26 (1.01,5.07) 0.048 | 2.11 (1.06,4.23) 0.034 |

After adjusting for age, sex, total charges, length of stay, admission types, rehab transfer, same day events, resident, insurance status, income, location of patient’s residence, disposition of patient and total comorbidities.

CML, chronic myeloid leukemia; MHNW, metabolically healthy normal weight; MUNW, metabolically unhealthy normal weight; MHO, metabolically healthy obesity; MUO, metabolically unhealthy obesity; NR, non-remission; aOR, adjusted odds ratio; CI, [confidence](javascript:;) [interval](javascript:;).

**Supplementary Table 12.** **Association of specific metabolic risk factors with the risk of adverse outcomes and disease burden in patients with CML in 30 days.**

|  | **NR/Relapse** | **Severe mortality risk** | **Disease burden** |
| --- | --- | --- | --- |
| **Variable** | **aOR (95% CI) P value** | **aOR (95% CI) P value** | **aOR (95% CI) P value** |
| Normal weight, no risk | 1 (Reference) | 1 (Reference) | 1 (Reference) |
| Normal weight, hyperglycemia | 1.68 (1.30,2.17) < 0.001 | 1.98 (1.45,2.68) < 0.001 | 1.74(1.36,2.22) < 0.001 |
| Normal weight, hypertension | 1.23 (1.06,1.42) 0.006 | 1.19 (0.98,1.43) 0.074 | 1.24 (1.08,1.43) 0.003 |
| Normal weight, hyperlipidemia | 0.84 (0.64,1.11) 0.210 | 1.04 (0.75,1.43) 0.831 | 0.92 (0.71,1.18) 0.507 |
| Obesity, no risk | 1.14 (0.84,1.54) 0.395 | 0.88 (0.55,1.40) 0.580 | 1.11 (0.83,1.48) 0.497 |
| Obesity, hyperglycemia | 0.98 (0.60,1.60) 0.918 | 1.64 (0.94,2.86) 0.082 | 1.20 (0.77,1.88) 0.419 |
| Obesity, hypertension | 1.52 (1.19,1.94) 0.01 | 1.41 (1.03,1.94) 0.033 | 1.43 (1.13,1.81) 0.003 |
| Obesity, hyperlipidemia | 0.98 (0.46,2.08) 0.962 | 2.49 (1.23,5.04) 0.011 | 2.04 (1.11,3.76) 0.022 |

After adjusting for age, sex, total charges, length of stay, admission types, rehab transfer, same day events, resident, insurance status, income, location of patient’s residence, disposition of patient and total comorbidities.

CML, chronic myeloid leukemia; MHNW, metabolically healthy normal weight; MUNW, metabolically unhealthy normal weight; MHO, metabolically healthy obesity; MUO, metabolically unhealthy obesity; NR, non-remission; aOR, adjusted odds ratio; CI, [confidence](javascript:;) [interval](javascript:;).

**Supplementary Table 13. Association of metabolically defined obesity with the risk of the severity of illness in patients with CML.**

|  | **Major loss of function in 30-day readmission** | **Major loss of function in 90-day readmission** | **Major loss of function in 180-day readmission** |
| --- | --- | --- | --- |
| **Variable** | **aOR (95% CI)** | **aOR (95% CI)** | **aOR (95% CI)** |
| MHNW | 1 (Reference) | 1 (Reference) | 1 (Reference) |
| MUNW | 1.29 (1.16, 1.42) <0.001 | 1.32 (1.18, 1.47) <0.001 | 1.32 (1.17, 1.49) <0.001 |
| MHO | 1.36 (1.13, 1.64) 0.001 | 1.33 (1.09, 1,61) 0.005 | 1.36 (1.09, 1.70) 0.007 |
| MUO | 1.87 (1.62, 2.17) <0.001 | 1.88 (1.61, 2.19) <0.001 | 1.79 (1.50, 2.14) <0.001 |

After adjusting for age, sex, total charges, length of stay, admission types, rehab transfer, same day events, resident, insurance status, income, location of patient’s residence, disposition of patient and total comorbidities.

CML, chronic myeloid leukemia; MHNW, metabolically healthy normal weight; MUNW, metabolically unhealthy normal weight; MHO, metabolically healthy obesity; MUO, metabolically unhealthy obesity; NR, non-remission; aOR, adjusted odds ratio; CI, [confidence](javascript:;) [interval](javascript:;).
